# Supplementary material for: Dietary-Induced Bacterial Metabolites Reduce Inflammation and Inflammation-Associated Cancer via Vitamin D Pathway
Source: Int J Mol Sci. 2023 Jan 18;24(3):1864. doi: 10.3390/ijms24031864 (PMC9914969; doi:10.3390/ijms24031864)
Supplement: Supplementary file 1 [file ijms-24-01864-s001.zip › ijms-1822471-supplementary/ijms-1822471 OMahony et al Suppl Tables revised.pdf]

Supplementary Table S1 – Diet composition

	Low fat diet (D12450H)		High fat diet (D12451)	
**Energy**	**Grams %**	**Kcal %**	**Grams %**	**Kcal %**
Protein	19.2	20	24	20
Carbohydrate	67.3	70	41	35
Fat	4.3	10	24	45
Total		*100*		*100*
Kcal/gm	*3.85*		*4.7*	
**Ingredients**	**Grams**	**Kcal**	**Grams**	**Kcal**
Casein, lactic, 30 Mesh	200	800	200	800
L-cysteine	3	12	3	12
Corn starch	452.2	1808.8	72.8	291
Maltodextrin10	75	300	100	400
Sucrose, fine granulated	172.8	691	172.8	691
Cellulose BW200	50	0	50	0
Soybean oil	25	225	25	225
Lard*	20	180	177.5	1598
Mineral Mix S10026	10	0	10	0
Dicalcium phosphate	13	0	13	0
Calcium Carbonate	5.5	0	5.5	0
Potassium citrate, 1H2O	16.5	0	16.5	0
Vitamin mix V10001	10	40	10	40
Choline bitartatre	2	0	2	0
FD&C yellow dye 5	0.04	0	0	0
FD&C red dye 40	0.01	0	0.05	0

* Cholesterol = 51.6 mg/gram

Supplementary Table S2 - mAbs used in flow cytometry

Target Molecule	Clone	Isotype	Conjugate	Source
**CD3**	145-2C11	IgG1	PE or PE Cy7	Biolegend
**CD49b**	DX5	IgM	APC	Biolegend
**CD69**	H1.2F3	IgG	PE Cy7	Biolegend
**CD4**	GK1.5	IgG2b, κ	PE	BD Biosciences
**Foxp3**	FJK-16s	IgG1a	APC	eBiosciences
**CD8**	53-6.7	IgG2a, κ	FITC	Biolegend
**CD11c**	HL3	IgG1, λ2	PE	BD Biosciences
**CD19**	6D5	IgG2a, κ	APC	Biolegend
**F4 80**	BM8	IgG2a, κ	FITC	Biolegend
**Ly6G**	RB6-8C5	IgG2b, κ	FITC	BD Biosciences
**MHC II**	AF6-120.1	IgG2a, κ	APC	Biolegend
**CD86**	GL1	IgG2a, κ	FITC	BD Biosciences

Supplementary Table S3 – Bile acids used in the study

Number	Analyte	$[MW]^{-H}$	Neutral Formula	RT	R_2 Value
1	Taurine	124.0068	$C_2H_7NO_3S$	0.75	0.962082
2	Dehydrocholic Acid	401.2328	$C_{24}H_{34}O_5$	2.00	0.986751
3	Lithocholic Acid	375.2899	$C_{24}H_{40}O_3$	21.72	0.968442
4	Hyodeoxycholic Acid	391.2848	$C_{24}H_{40}O_4$	9.40	0.994262
5	Chenodeoxycholic Acid	391.2848	$C_{24}H_{40}O_4$	18.38	0.995447
6	Ursodeoxycholic aAid	391.2848	$C_{24}H_{40}O_4$	11.97	0.994563
7	Deoxycholic Acid	391.2848	$C_{24}H_{40}O_4$	19.13	0.9897113
8	Cholic Acid	407.2797	$C_{24}H_{40}O_5$	13.01	0.894385
9	Hyocholic Acid	407.2797	$C_{24}H_{40}O_5$	11.39	0.993712
10	Taurocholic Acid	514.2838	$C_{26}H_{45}NO_7S$	5.29	0.993712
11	Taurohyocholic Acid	514.2838	$C_{26}H_{45}NO_7S$	2.12	0.992805
12	Taurochenodeoxycholic Acid	498.2889	$C_{26}H_{45}NO_6S$	8.99	0.991546
13	Tauroursodeoxycholic Acid	498.2889	$C_{26}H_{45}NO_6S$	3.20	0.988966
14	Taurodeoxycholic Acid	498.2889	$C_{26}H_{45}NO_6S$	10.27	0.991519
15	Taurohyodeoxycholic Acid	498.2889	$C_{26}H_{45}NO_6S$	3.76	0.987378
16	Taurolithocholic Acid	482.2940	$C_{26}H_{45}NO_5S$	14.61	0.988162
17	α-Muricholic Acid	407.2797	$C_{24}H_{40}O_5$	6.52	0.995697
18	β-Muricholic Acid	407.2797	$C_{24}H_{40}O_5$	6.93	0.998331
19	ω-Muricholic Acid	407.2797	$C_{24}H_{40}O_5$	5.80	0.978579
20	Tauro α-Muricholic Acid	514.2838	$C_{26}H_{45}NO_7S$	1.63	0.995563
21	Tauro β-Muricholic Acid	514.2838	$C_{26}H_{45}NO_7S$	1.19	0.980216
22	Tauro ω-Muricholic Acid	514.2838	$C_{26}H_{45}NO_7S$	1.80	0.988878
D1	Cholic Acid d4	411.3049	$C_{24}H_{36}D_4O_5$	13.01	NA
D2	Chenodeoxycholic Acid d4	395.3099	$C_{24}H_{36}D_4O_4$	18.31	NA

Supplementary Table S4 – Faecal microbiota composition in Control groups in the Chronic-Colitis and Colitis-associated cancer (CAC) study

a) Phylum

	Week 5						Week 7						Week 15					
	LF		HF				LF		HF				LF		HF			
	Mean	Median	Mean	Median	p-value	Adj p-value	Mean	Median	Mean	Median	p-value	Adj p-value	Mean	Median	Mean	Median	p-value	Adj p-value
Actinobacteria	13.10	15.05	3.96	2.48	0.007##	0.021*	4.81	5.50	3.04	0.61	0.399	0.565	1.60	1.11	1.83	1.79	0.798	0.798
Bacteroidetes	23.05	22.82	35.75	38.51	0.161	0.234	30.55	34.36	20.70	19.51	0.108	0.216	33.77	29.69	29.25	21.90	0.328	0.606
Deferribacteres	0.86	0.33	3.76	1.57	0.195	0.234	0.91	0.16	0.19	0.18	0.651	0.651	1.69	1.44	1.98	0.43	0.505	0.606
Firmicutes	61.73	62.05	56.31	56.75	0.382	0.382	62.94	59.63	76.00	75.65	0.043	0.128	61.47	66.49	66.84	71.14	0.442	0.606
Proteobacteria	1.23	1.36	0.10	0.00	0.002##	0.01**	0.78	0.66	0.08	0.03	0.004##	0.021*	1.43	0.49	0.00	0.00	0.0004###	0.002**
unclassified	0.03	0.00	0.12	0.10	0.178	0.234	0.01	0.00	0.00	0.00	0.470	0.565	0.04	0.00	0.11	0.07	0.217	0.606

p-value: # p <0.05, ## p < 0.01; ### p < 0.001 - LF-fed control mice compared to HF-fed control mice

Adjusted p-value: * p <0.05, ** p < 0.01 - LF-fed control mice compared to HF-fed control mice. P-values were adjusted for multiple testing using the Benjamini-Hochberg method

b) Family

	Week 5						Week 7						Week 15					
	LF		HF				LF		HF				LF		HF			
	Mean	Median	Mean	Median	p-value	Adj p-value	Mean	Median	Mean	Median	p-value	Adj p-value	Mean	Median	Mean	Median	p-value	Adj p-value
Bacteroidaceae	3.76	3.19	11.86	8.00	0.161	0.353	4.24	5.07	8.33	6.67	0.228	0.542	8.44	7.19	9.76	5.40	0.798	0.878
Bifidobacteriaceae	13.10	15.05	3.96	2.48	0.007##	0.019*	4.81	5.50	3.04	0.61	0.399	0.549	1.60	1.11	1.83	1.79	0.798	0.878
Deferribacteraceae	0.86	0.33	3.76	1.57	0.195	0.357	0.91	0.16	0.19	0.18	0.651	0.728	1.69	1.44	1.98	0.43	0.505	0.878
Erysipelotrichaceae	52.40	52.95	29.10	31.50	0.003##	0.011*	51.22	48.13	58.91	60.98	0.345	0.542	47.05	41.82	43.79	49.42	0.798	0.878
Lachnospiraceae	4.38	4.53	13.93	14.39	0.001###	0.006**	6.24	5.49	4.56	3.71	0.345	0.542	8.04	7.06	12.86	12.12	0.442	0.878
Lactobacillaceae	2.96	1.47	4.65	4.38	0.382	0.526	2.48	1.51	5.01	3.54	0.345	0.542	1.87	1.69	6.08	1.92	0.645	0.878
Porphyromonadaceae	10.07	9.31	11.15	11.52	0.721	0.793	10.32	9.54	7.77	8.22	0.345	0.542	14.89	14.26	12.93	9.96	0.328	0.878
Rikenellaceae	7.73	7.98	9.37	7.52	0.878	0.878	14.44	15.44	2.79	3.15	0.0006###	0.007**	8.15	6.48	3.91	2.70	0.059	0.322
Ruminococcaceae	1.88	2.04	8.39	8.09	0.0002###	0.002**	2.88	2.85	7.35	7.15	0.02#	0.110	4.32	3.17	3.93	3.04	0.878	0.878
Sutterellaceae	0.26	0.15	0.10	0.00	0.351	0.526	0.06	0.00	0.07	0.03	0.775	0.775	0.89	0.03	0.00	0.00	0.032#	0.322
unclassified	2.61	2.19	3.73	2.62	0.645	0.789	2.39	2.06	1.98	1.76	0.662	0.728	3.09	2.82	2.93	2.56	0.721	0.878

p-value: # p <0.05, ## p < 0.01; ### p < 0.001 - LF-fed control mice compared to HF-fed control mice

Adjusted p-value: * p <0.05, ** p < 0.01 - LF-fed control mice compared to HF-fed control mice. P-values were adjusted for multiple testing using the Benjamini-Hochberg method

c) Genus

	Week 5						Week 7						Week 15					
	LF		HF				LF		HF				LF		HF			
	Mean	Median	Mean	Median	p-value	Adj p-value	Mean	Median	Mean	Median	p-value	Adj p-value	Mean	Median	Mean	Median	p-value	Adj p-value
Alistipes	7.73	7.98	9.37	7.52	0.878	0.878	14.44	15.44	2.79	3.15	0.0001###	0.012*	8.15	6.48	3.91	2.70	0.059	0.176
Allobaculum	52.39	52.95	27.71	30.49	0.002##	0.011*	51.22	48.13	58.46	59.78	0.345	0.677	47.05	41.82	41.72	45.58	0.574	0.861
Bacteroides	3.76	3.19	11.86	8.00	0.161	0.263	4.24	5.07	8.33	6.67	0.228	0.587	8.44	7.19	9.76	5.40	0.798	0.878
Barnesiella	1.53	1.45	2.60	2.51	0.234	0.325	1.90	1.91	3.15	2.21	0.755	0.930	4.34	3.72	4.09	2.43	0.130	0.335
Bifidobacterium	13.10	15.05	3.96	2.48	0.007##	0.025*	4.81	5.50	3.04	0.61	0.399	0.677	1.60	1.11	1.83	1.79	0.798	0.878
Clostridium_XlVa	0.02	0.00	1.40	0.45	0.002##	0.011*	0.11	0.00	0.83	0.87	0.007##	0.061	0.26	0.07	0.75	0.79	0.023#	0.117
Dorea	0.43	0.41	1.14	0.85	0.010#	0.027*	0.52	0.40	0.78	0.88	0.414	0.677	0.46	0.33	0.46	0.23	0.878	0.878
Flavonifractor	0.49	0.44	1.05	0.84	0.093	0.167	0.33	0.30	1.58	0.45	0.755	0.930	0.28	0.14	0.26	0.13	0.875	0.878
Johnsonella	0.01	0.00	0.22	0.09	0.03#	0.067	0.03	0.00	0.13	0.06	0.133	0.400	0.10	0.04	0.03	0.00	0.305	0.668
Lactobacillus	2.96	1.47	4.65	4.38	0.382	0.405	2.48	1.51	5.01	3.54	0.345	0.677	1.87	1.69	6.08	1.92	0.645	0.878
Mucispirillum	0.86	0.33	3.76	1.57	0.195	0.292	0.91	0.16	0.19	0.18	0.651	0.930	1.69	1.44	1.98	0.43	0.505	0.861
Odoribacter	0.47	0.49	1.35	0.70	0.328	0.394	0.53	0.57	0.99	0.34	1.000	1.000	0.70	0.90	0.58	0.47	0.574	0.861
Oscillibacter	0.43	0.40	1.89	1.91	0.0001###	0.011*	0.74	0.43	2.03	1.80	0.059	0.267	0.70	0.55	1.29	1.25	0.028#	0.117
Parabacteroides	0.51	0.46	0.36	0.16	0.051	0.103	0.46	0.31	0.32	0.30	0.852	0.958	3.15	2.75	0.93	0.25	0.007##	0.063
Parasutterella	0.26	0.15	0.10	0.00	0.351	0.395	0.06	0.00	0.07	0.03	0.775	0.930	0.89	0.03	0.00	0.00	0.032#	0.117
Sporobacter	0.05	0.02	0.11	0.06	0.298	0.383	0.01	0.00	0.06	0.00	0.928	0.983	0.01	0.00	0.03	0.00	0.334	0.668
Turicibacter	0.01	0.00	1.39	1.01	0.002##	0.011*	0.00	0.00	0.44	0.06	0.039#	0.233	0.00	0.00	2.07	0.62	0.005##	0.063
unclassified	15.00	16.08	27.08	28.04	0.010#	0.027*	17.21	16.06	11.80	11.14	0.108	0.388	20.32	18.54	24.22	21.06	0.798	0.878

p-value: # p <0.05, ## p < 0.01; ### p < 0.001 - LF-fed control mice compared to HF-fed control mice

Adjusted p-value: * p <0.05 - LF-fed control mice compared to HF-fed control mice. P-values were adjusted for multiple testing using the Benjamini-Hochberg method

Supplementary Table S5 – Faecal microbiota composition in Chronic-Colitis groups

a) Phylum

	Week 5						Week 7						Week 15					
	LF		HF				LF		HF				LF		HF			
	**Mean**	**Median**	**Mean**	**Median**	**p-value**	**Adj p-value**	**Mean**	**Median**	**Mean**	**Median**	**p-value**	**Adj p-value**	**Mean**	**Median**	**Mean**	**Median**	**p-value**	**Adj p-value**
Actinobacteria	2.35	1.10	5.13	1.45	0.840	0.840	0.45	0.12	3.89	0.40	0.148	0.178	1.02	1.13	3.27	2.47	0.056	0.167
Bacteroidetes	23.57	21.28	29.58	30.11	0.351	0.702	44.81	44.26	29.59	32.29	0.020#	0.078	29.74	28.92	31.70	40.18	0.659	0.930
Deferribacteres	6.91	5.99	7.29	4.62	0.778	0.840	0.42	0.20	0.66	0.73	0.534	0.534	4.98	2.56	6.14	2.50	0.930	0.930
Firmicutes	65.77	65.03	57.32	60.31	0.238	0.702	43.10	40.36	60.24	59.48	0.026#	0.078	54.43	62.02	55.14	51.92	0.930	0.930
Proteobacteria	1.34	1.02	0.60	0.37	0.109	0.652	11.21	10.34	5.59	4.17	0.062	0.124	9.78	8.78	3.66	2.64	0.046	0.167
unclassified	0.06	0.05	0.08	0.05	0.764	0.840	0.00	0.00	0.04	0.00	0.118	0.177	0.04	0.00	0.08	0.00	0.359	0.718

p-value: # p <0.05, ## p < 0.01; ### p < 0.001 - LF-fed colitis mice compared to HF-fed colitis mice
Adjusted p-value: * p <0.05, ** p < 0.01 - LF-fed colitis mice compared to HF-fed colitis mice. P-values were adjusted for multiple testing using the Benjamini-Hochberg method

b) Family

	Week 5						Week 7						Week 15					
	LF		HF				LF		HF				LF		HF			
	**Mean**	**Median**	**Mean**	**Median**	**p-value**	**Adj p-value**	**Mean**	**Median**	**Mean**	**Median**	**p-value**	**Adj p-value**	**Mean**	**Median**	**Mean**	**Median**	**p-value**	**Adj p-value**
Bacteroidaceae	6.41	4.28	14.31	7.57	0.272	0.499	30.93	34.64	18.89	20.91	0.020#	0.075	14.68	13.56	18.46	19.63	0.328	0.516
Bifidobacteriaceae	2.35	1.10	5.13	1.45	0.840	0.840	0.45	0.12	3.89	0.40	0.148	0.326	1.02	1.13	3.27	2.47	0.056	0.242
Deferribacteraceae	6.91	5.99	7.29	4.62	0.778	0.840	0.42	0.20	0.66	0.73	0.534	0.599	4.98	2.56	6.14	2.50	0.930	0.930
Erysipelotrichaceae	40.28	33.74	37.72	35.10	0.840	0.840	37.69	34.55	44.05	42.87	0.442	0.599	37.54	36.67	42.52	41.74	0.659	0.725
Lachnospiraceae	15.99	13.22	11.44	6.31	0.177	0.474	0.77	0.50	8.34	7.52	0.00003###	0.0003***	13.33	6.99	7.48	3.92	0.211	0.452
Lactobacillaceae	4.46	3.39	2.51	1.43	0.215	0.474	4.08	1.76	1.93	1.27	0.657	0.657	0.68	0.10	1.83	0.80	0.067	0.242
Porphyromonadacea	10.39	10.74	6.11	5.23	0.009##	0.100	10.58	10.48	8.86	8.23	0.545	0.599	11.91	10.98	6.39	5.32	0.044#	0.242
Rikenellaceae	5.01	3.58	7.25	7.45	0.091	0.474	2.66	1.75	1.25	0.74	0.247	0.428	2.48	1.99	5.45	2.10	0.246	0.452
Ruminococcaceae	4.77	4.72	5.56	6.69	0.545	0.749	0.56	0.40	5.73	3.75	0.002##	0.011*	2.65	1.72	3.17	3.10	0.536	0.655
Sutterellaceae	0.74	0.38	0.43	0.14	0.184	0.474	5.50	3.58	1.98	1.09	0.090	0.249	5.12	3.78	0.76	0.09	0.088	0.242
unclassified	2.68	1.79	2.25	1.81	0.395	0.621	6.36	4.64	4.42	2.57	0.272	0.428	5.60	3.84	4.52	4.35	0.536	0.655

p-value: # p <0.05, ## p < 0.01; ### p < 0.001 - LF-fed colitis mice compared to HF-fed colitis mice
Adjusted p-value: * p <0.05, *** p < 0.001 - LF-fed colitis mice compared to HF-fed colitis mice. P-values were adjusted for multiple testing using the Benjamini-Hochberg method

c) Genus

	Week 5						Week 7						Week 15					
	LF		HF				LF		HF				LF		HF			
	Mean	Median	Mean	Median	p-value	Adj p-value	Mean	Median	Mean	Median	p-value	Adj p-value	Mean	Median	Mean	Median	p-value	Adj p-value
Alistipes	5.01	3.58	7.25	7.45	0.091	0.409	2.66	1.75	1.25	0.74	0.247	0.343	2.48	1.99	5.45	2.10	0.246	0.419
Allobaculum	40.16	33.64	37.66	35.10	0.840	0.890	37.59	34.55	44.02	42.82	0.442	0.568	37.54	36.67	42.47	41.74	0.659	0.747
Bacteroides	6.41	4.28	14.31	7.57	0.272	0.570	30.93	34.64	18.89	20.91	0.020[#]	0.073	14.68	13.56	18.46	19.63	0.328	0.505
Barnesiella	1.20	1.06	2.16	1.79	0.075	0.409	4.87	4.13	3.86	2.93	0.600	0.635	3.05	2.20	2.14	1.69	0.479	0.582
Bifidobacterium	2.35	1.10	5.13	1.45	0.840	0.890	0.45	0.12	3.89	0.40	0.148	0.248	1.02	1.13	3.27	2.47	0.056	0.295
Clostridium_XlVa	0.04	0.00	0.77	0.05	0.348	0.570	0.04	0.00	0.34	0.17	0.013[#]	0.071	0.39	0.15	1.11	0.33	0.147	0.357
Dorea	0.90	0.83	0.99	0.89	0.717	0.890	0.07	0.00	0.46	0.38	0.010[#]	0.071	0.39	0.38	0.17	0.16	0.234	0.419
Flavonifractor	0.54	0.29	0.37	0.19	0.429	0.643	0.04	0.00	0.32	0.10	0.198	0.297	0.49	0.30	0.13	0.08	0.356	0.505
Johnsonella	0.08	0.06	0.05	0.00	0.089	0.409	0.00	0.00	0.06	0.00	0.104	0.208	0.20	0.00	0.01	0.00	0.456	0.582
Lactobacillus	4.46	3.39	2.51	1.43	0.215	0.570	4.08	1.76	1.93	1.27	0.657	0.657	0.68	0.10	1.83	0.80	0.067	0.295
Mucispirillum	6.91	5.99	7.29	4.62	0.778	0.890	0.42	0.20	0.66	0.73	0.534	0.601	4.98	2.56	6.14	2.50	0.930	0.930
Odoribacter	0.49	0.29	0.40	0.29	0.967	0.967	0.00	0.00	0.04	0.00	0.104	0.208	0.00	0.00	0.00	0.00	NA	NA
Oscillibacter	1.10	1.18	1.31	1.46	0.492	0.681	0.16	0.00	0.74	0.49	0.008[##]	0.071	0.70	0.37	0.92	1.21	0.205	0.419
Parabacteroides	2.28	1.41	0.28	0.25	0.004[##]	0.079	2.25	2.16	1.29	1.07	0.152	0.248	3.28	2.08	0.78	0.66	0.085	0.295
Parasutterella	0.74	0.38	0.43	0.14	0.184	0.570	5.50	3.58	1.98	1.09	0.090	0.208	5.12	3.78	0.76	0.09	0.088	0.295
Sporobacter	0.03	0.00	0.22	0.00	0.287	0.570	0.00	0.00	0.08	0.00	0.038[#]	0.113	0.05	0.00	0.05	0.00	0.889	0.930
Turicibacter	0.12	0.00	0.06	0.00	0.338	0.570	0.10	0.00	0.02	0.00	0.475	0.570	0.00	0.00	0.05	0.00	0.082	0.295
unclassified	27.17	26.31	18.81	11.47	0.238	0.570	10.83	10.50	20.16	18.24	0.016[#]	0.071	24.95	25.67	16.25	18.81	0.104	0.295

p-value: # $p < 0.05$, ## $p < 0.01$ - LF-fed colitis mice compared to HF-fed colitis mice
NA – not applicable

Supplementary Table S6 – Faecal microbiota composition in Colitis associated cancer (CAC) groups

a) Phylum

	Week 5						Week 7						Week 15					
	LF		HF				LF		HF				LF		HF			
	Mean	Median	Mean	Median	p-value	Adj p-value	Mean	Median	Mean	Median	p-value	Adj p-value	Mean	Median	Mean	Median	p-value	Adj p-value
Actinobacteria	4.30	3.23	7.84	6.19	0.183	0.219	1.01	0.25	1.59	1.93	0.558	0.836	1.03	0.68	2.48	2.44	0.079	0.158
Bacteroidetes	33.79	33.11	21.60	18.89	0.021#	0.062	44.64	42.63	20.91	19.06	0.045#	0.090	43.57	39.91	32.67	25.97	0.315	0.473
Deferribacteres	6.25	4.67	1.07	0.66	0.152	0.219	2.38	1.15	3.33	2.19	0.724	0.869	3.35	0.67	2.04	0.30	0.414	0.497
Firmicutes	53.69	54.13	68.79	66.32	0.009###	0.056	42.58	43.14	71.33	74.04	0.006##	0.037*	41.39	46.43	59.23	55.95	0.079	0.158
Proteobacteria	1.96	1.34	0.69	0.42	0.121	0.219	9.38	8.19	2.83	1.17	0.019#	0.056	10.61	9.75	3.56	2.12	0.004##	0.025*
unclassified	0.00	0.00	0.01	0.00	0.350	0.350	0.01	0.00	0.01	0.00	0.907	0.907	0.05	0.00	0.02	0.00	0.674	0.674

p-value: # p <0.05, ## p < 0.01; ### p < 0.001 - LF-fed CAC mice compared to HF-fed CAC mice
Adjusted p-value: * p <0.05 - LF-fed CAC mice compared to HF-fed CAC mice. P-values were adjusted for multiple testing using the Benjamini-Hochberg method

b) Family

	Week 5						Week 7						Week 15					
	LF		HF				LF		HF				LF		HF			
	Mean	Median	Mean	Median	p-value	Adj p-value	Mean	Median	Mean	Median	p-value	Adj p-value	Mean	Median	Mean	Median	p-value	Adj p-value
Bacteroidaceae	14.08	13.59	9.81	6.41	0.336	0.462	34.91	33.44	11.88	12.45	0.006##	0.034*	27.07	26.19	17.81	14.10	0.315	0.546
Bifidobacteriaceae	4.30	3.23	7.84	6.19	0.183	0.297	1.01	0.25	1.59	1.93	0.558	0.767	1.03	0.68	2.48	2.44	0.079	0.289
Deferribacteraceae	6.25	4.67	1.07	0.66	0.152	0.297	2.38	1.15	3.33	2.19	0.724	0.846	3.35	0.67	2.04	0.30	0.414	0.546
Erysipelotrichaceae	43.35	45.20	52.92	52.15	0.189	0.297	33.80	31.29	57.03	57.87	0.065	0.239	33.01	31.86	52.63	53.23	0.053	0.289
Lachnospiraceae	5.69	3.85	5.77	4.51	0.779	0.857	3.43	2.43	6.81	5.08	0.284	0.626	5.47	2.43	2.64	2.32	0.780	0.858
Lactobacillaceae	1.81	0.39	6.21	4.95	0.043#	0.156	2.53	0.96	1.54	1.36	1.000	1.000	0.22	0.07	1.26	0.34	0.281	0.546
Porphyromonadaceae	10.53	10.51	6.97	6.76	0.029#	0.156	7.99	9.39	6.95	6.96	0.524	0.767	11.51	10.63	8.49	9.11	0.447	0.546
Rikenellaceae	7.62	8.48	4.06	4.66	0.094	0.258	1.29	0.66	1.84	1.16	0.093	0.256	4.16	2.74	5.36	2.15	0.968	0.968
Ruminococcaceae	2.52	1.78	3.78	2.31	0.5358	0.655	2.66	2.27	5.81	7.02	0.354	0.650	2.35	1.61	2.50	2.56	0.356	0.546
Sutterellaceae	1.19	0.49	0.62	0.33	0.862	0.862	3.83	2.22	2.65	1.06	0.769	0.846	2.81	3.21	0.44	0.08	0.258	0.546
unclassified	2.64	2.25	0.95	0.98	0.0003###	0.003**	6.17	6.46	0.57	0.51	0.0016##	0.017*	9.02	8.47	4.35	3.19	0.017#	0.189

p-value: # p <0.05, ## p < 0.01; ### p < 0.001 - LF-fed CAC mice compared to HF-fed CAC mice
Adjusted p-value: * p <0.05 - LF-fed CAC mice compared to HF-fed CAC mice. P-values were adjusted for multiple testing using the Benjamini-Hochberg method

c) Genus

	**Week 5**						**Week 7**						**Week 15**					
	**LF**		**HF**				**LF**		**HF**				**LF**		**HF**			
	**Mean**	**Median**	**Mean**	**Median**	**p-value**	**Adj p-value**	**Mean**	**Median**	**Mean**	**Median**	**p-value**	**Adj p-value**	**Mean**	**Median**	**Mean**	**Median**	**p-value**	**Adj p-value**
Alistipes	7.62	8.48	4.06	4.66	0.094	0.252	1.29	0.66	1.84	1.16	0.093	0.226	4.16	2.74	5.36	2.15	0.968	0.968
Allobaculum	43.18	45.15	52.92	52.15	0.189	0.310	33.32	31.18	57.03	57.87	0.065	0.185	33.01	31.86	52.63	53.23	0.053	0.224
Bacteroides	14.08	13.59	9.81	6.41	0.336	0.503	34.91	33.44	11.88	12.45	$0.006^{\#\#}$	0.035*	27.07	26.19	17.81	14.10	0.315	0.447
Barnesiella	3.07	2.61	2.10	1.79	0.613	0.788	3.59	3.41	3.42	2.99	1.000	1.000	3.89	3.42	3.63	2.16	0.604	0.642
Bifidobacterium	4.30	3.23	7.84	6.19	0.183	0.310	1.01	0.25	1.59	1.93	0.558	0.948	1.03	0.68	2.48	2.44	0.079	0.224
Clostridium_XlVa	0.04	0.00	0.50	0.41	$0.009^{\#\#}$	0.079	0.01	0.00	0.22	0.11	$0.003^{\#\#}$	0.035*	0.12	0.00	0.24	0.24	0.162	0.342
Dorea	0.53	0.56	0.50	0.33	1.000	1.000	0.24	0.19	0.21	0.14	1.000	1.000	0.16	0.00	0.15	0.09	0.517	0.612
Flavonifractor	0.15	0.00	0.54	0.42	0.120	0.271	0.25	0.12	0.52	0.51	0.711	1.000	0.27	0.19	0.09	0.08	$0.008^{\#\#}$	0.075
Johnsonella	0.08	0.05	0.33	0.11	0.428	0.592	0.00	0.00	0.93	0.11	$0.006^{\#\#}$	0.035*	0.45	0.00	0.20	0.22	0.181	0.342
Lactobacillus	1.81	0.39	6.21	4.95	$0.043^{\#}$	0.192	2.53	0.96	1.54	1.36	1.000	1.000	0.22	0.07	1.26	0.34	0.281	0.434
Mucispirillum	6.25	4.67	1.07	0.66	0.152	0.304	2.38	1.15	3.33	2.19	0.724	1.000	3.35	0.67	2.04	0.30	0.414	0.541
Odoribacter	0.52	0.37	0.09	0.00	$0.036^{\#}$	0.192	0.00	0.00	0.00	0.00	NA	NA	0.00	0.00	0.03	0.00	0.146	0.342
Oscillibacter	0.55	0.45	0.50	0.44	0.955	1.000	0.33	0.21	0.80	0.64	0.164	0.348	0.55	0.35	0.54	0.32	0.540	0.612
Parabacteroides	1.58	1.09	0.38	0.33	$0.006^{\#\#}$	0.079	2.60	1.69	0.50	0.17	$0.019^{\#}$	0.079	4.19	2.57	1.10	0.52	$0.013^{\#}$	0.075
Parasutterella	1.19	0.49	0.62	0.33	0.862	0.970	3.83	2.22	2.65	1.06	0.769	1.000	2.81	3.21	0.44	0.08	0.258	0.434
Sporobacter	0.01	0.00	0.08	0.08	0.073	0.252	0.00	0.00	0.01	0.00	0.268	0.507	0.00	0.00	0.09	0.06	$0.010^{\#\#}$	0.075
Turicibacter	0.17	0.00	0.00	0.00	0.098	0.252	0.48	0.11	0.00	0.00	$0.045^{\#}$	0.154	0.00	0.00	0.00	0.00	NA	NA
unclassified	14.85	12.58	12.46	11.23	0.694	0.833	13.23	12.95	13.54	12.30	0.833	1.000	18.72	14.79	11.90	9.93	0.079	0.224

p-value: # p <0.05, ## p < 0.01; ### p < 0.001 - LF-fed CAC mice compared to HF-fed CAC mice
Adjusted p-value: * p <0.05 - LF-fed CAC mice compared to HF-fed CAC mice. P-values were adjusted for multiple testing using the Benjamini-Hochberg method
NA – not applicable

Supplementary Table S7 – Metabolic markers in mice fed either high fat (HF) or low fat (LF) diets either alone or challenged with DSS (Colitis) and AOM and DSS (CAC)

	AOM-LF-DSS [a]	AOM-HF-DSS [a]	LF-AOM-DSS [b]	HF-AOM-DSS [b]	LF-AOM [c]	HF-AOM [c]
**% Body Fat**	13.4±1.0	31.7±1.6 ***	12.2±0.7	25.3±2.2 ###	19.9±2.5	32.2±5.5
**% Lean**	69.4±1.0	53.0±1.4 ***	69.9±0.6	58.2±1.9 ###	62.4±2.2	52.9±4.9
**Colon length (cm)**	5.4±0.2	6.0±0.2 *	6.0 ± 0.3	6.1±0.1	6.6±0.7	6.9±0.3
**Colon weight (mg/cm)**	72.7±9.7	45.0±3.0 *	56.8±4.6	42.1±3.6 ##	29.8±5.3	26.7±1.6
**Tumor number/mouse**	11.9±2.4	2.3±0.8 **	4.5±1.1	1.9±0.4 #	1±0.4	2.3±0.9 &

[a]AOM-LF-DSS and AOM-HF-DSS groups - Mice were injected intraperitoneally with AOM followed by feeding with LF and HF diet and exposed to 3 cycles of DSS, 5 days 1.5%DSS and 14 days of water

[b] LF-AOM-DSS and HF-AOM-DSS groups - Mice were fed LF and HF diet followed by intraperitoneal injection of azoxymethane (AOM) and exposed to 3 cycles of DSS, 5 days 1.5%DSS and 14 days of water

[c] AOM-LF and AOM-HF groups - Mice were fed LF and HF diet followed by intraperitoneal injection of AOM

All values are means ± SEMs. n=3-4/group for AOM-treated mice and 9-10 mice/group of AOM-DSS-treated mice.

* p <0.0.5 and ** p <0.01 and *** p <0.001 for differences between AOM-HF-DSS vs AOM-LF-DSS, (ANOVA followed by post hoc correction)

# p < 0.05 and ## p < 0.01 and ### p < 0.001 for differences between HF-AOM-DSS vs LF-AOM-DSS, (ANOVA followed by post hoc correction)

& p =0.09 between HF-AOM vs LF-AOM, (Two-tail unpaired Student's *t*-test)

Supplementary Table S8. Fatty acid profile in the liver of mice fed either high fat (HF) or low fat (LF) diets either alone or challenged with DSS (Colitis) and AOM and DSS (CAC)

**Fatty Acid**	**LF Control (g/100g FAME)**	**LF Colitis (g/100g FAME)**	**LF CAC (g/100g FAME)**	**HF Control (g/100g FAME)**	**HF Colitis (g/100g FAME)**	**HF CAC (g/100g FAME)**
myristic acid/C14:0	0.42 ± 0.03	0.42 ± 0.02	0.43 ± 0.02	0.39 ± 0.02[a]	0.39 ± 0.02[a]	0.22 ± 0.02[b]
palmitic acid/C16:0	23.92 ± 0.89	24.38 ± 0.47	24.77 ± 0.62	24.95 ± 0.36[a]	24.31 ± 0.40[a]	22.96 ± 0.37[b]
palmitoleic acid/C16:1c9	2.74 ± 0.14	2.92 ± 0.12	2.86 ± 0.22	2.26 ± 0.19[a]	1.57 ± 0.20[b]	1.48 ± 0.14[b]
Stearic acid/C18:0	11.99 ± 0.31[####]	10.75 ± 0.50	11.22 ± 0.82	5.03 ± 0.49[a]	10.20 ± 1.24[b]	8.34 ± 0.60[a,b]
oleic acid/C18:1c9	23.06 ± 0.49[a, ####]	27.62 ± 0.98[b]	26.19 ± 1.60[a,b]	36.86 ± 1.29[a]	27.64 ± 2.31[b]	30.85 ± 1.32[a,b]
linoleic acid /C18:2n-6	13.32 ± 0.32[a, #]	10.32 ± 0.36[b]	11.13 ± 0.42[b]	14.98 ± 0.51[a]	15.79 ± 0.46[a]	17.77 ± 0.29[b]

linolenic acid/C18:3n-3	0.35 ± 0.02[a, #]	0.27 ± 0.01[b]	0.30 ± 0.03[a,b]	0.27 ± 0.02[a]	0.34 ± 0.03[a,b]	0.38 ± 0.02[b]
γ-linoleic acid /C18:3n-6	0.20 ± 0.01[a, #]	0.14 ± 0.01[b]	0.17 ± 0.02[a,b]	0.27 ± 0.02	0.28 ± 0.02	0.29 ± 0.02
dihomo-γ-linolenic acid/ C20:3n-6	1.18 ± 0.05[a, ##]	1.04 ± 0.02[a,b]	0.94 ± 0.10[b]	0.99 ± 0.02	0.99 ± 0.02	1.03 ± 0.04
AA/C20:4n-6	9.72 ± 0.40[a, ####]	8.16 ± 0.27[b]	7.87 ± 0.52[b]	4.63 ± 0.52[a]	7.86 ± 0.82[b]	6.69 ± 0.39[a,b]
EPA/C20:5n-3	0.19 ± 0.01[a, ###]	0.13 ± 0.01[b]	0.13 ± 0.01[b]	0.12 ± 0.01	0.13 ± 0.01	0.11 ± 0.02
DPA/C22:5n-3	0.25 ± 0.02[a, ##]	0.15 ± 0.01[b]	0.23 ± 0.01[a]	0.34 ± 0.03	0.28 ± 0.01	0.32 ± 0.04
DHA/C22:6n-3	5.64 ± 0.24[a, ####]	4.32 ± 0.17[b]	4.37 ± 0.24[b]	2.51 ± 0.28	3.71 ± 0.38	3.23 ± 0.30

[a,b] Values in the same row which do not share a common superscript letter are significantly different, p < 0.05 (ANOVA followed by post hoc Tuckey's multiple comparisons tests)

All HF groups are compared to each other only and all LF groups are compared to each other only

#, ##, ###, #### represent p < 0.05, p < 0.01, p < 0.001 and p < 0.0001, respectively, when comparing LF vs HF control groups

arachidonic acid – AA; eicosapentaenoic acid - EPA; docosahexaenoic acid – DHA; docosapentaenoic acid – DPA

Red font – higher concentration of Fatty acid compared to respective Control.

Green font – lower concentration of Fatty acid compared to respective Control.
