# Supplementary material for: Dietary-Induced Bacterial Metabolites Reduce Inflammation and Inflammation-Associated Cancer via Vitamin D Pathway
Source: Int J Mol Sci. 2023 Jan 18;24(3):1864. doi: 10.3390/ijms24031864 (PMC9914969; doi:10.3390/ijms24031864)
Supplement: Supplementary file 1 [file ijms-24-01864-s001.zip › ijms-1822471-supplementary/ijms-1822471-Suppl figures revised SM.pptx]

## Slide 1
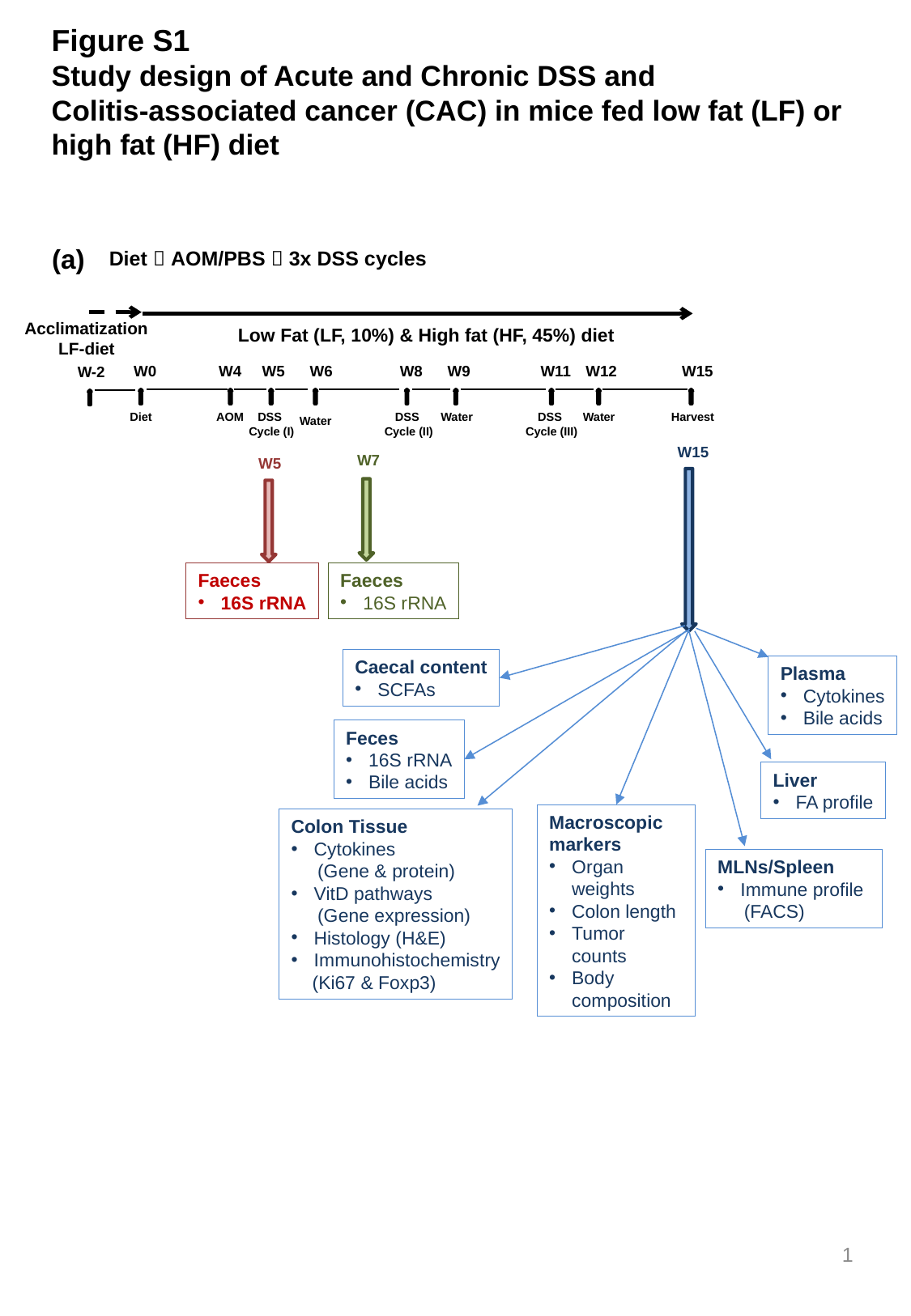

Figure S1
Study design of Acute and Chronic DSS and
Colitis-associated cancer (CAC) in mice fed low fat (LF) or high fat (HF) diet
(a)
Diet  AOM/PBS  3x DSS cycles
Acclimatization
LF-diet
Low Fat (LF, 10%) & High fat (HF, 45%) diet
W0
W4
W5
W6
W8
W9
W11
W12
W15
W-2
Diet
AOM
DSS
Cycle (I)
DSS
Cycle (II)
Water
DSS
Cycle (III)
Water
Harvest
Water
W15
W7
W5
Faeces
16S rRNA
Faeces
16S rRNA
Caecal content
SCFAs
Plasma
Cytokines
Bile acids
Feces
16S rRNA
Bile acids
Liver
FA profile
Macroscopic markers
Organ weights
Colon length
Tumor counts
Body composition
Colon Tissue
Cytokines
 (Gene & protein)
VitD pathways
 (Gene expression)
Histology (H&E)
Immunohistochemistry
 (Ki67 & Foxp3)
MLNs/Spleen
Immune profile
 (FACS)
1

## Slide 2
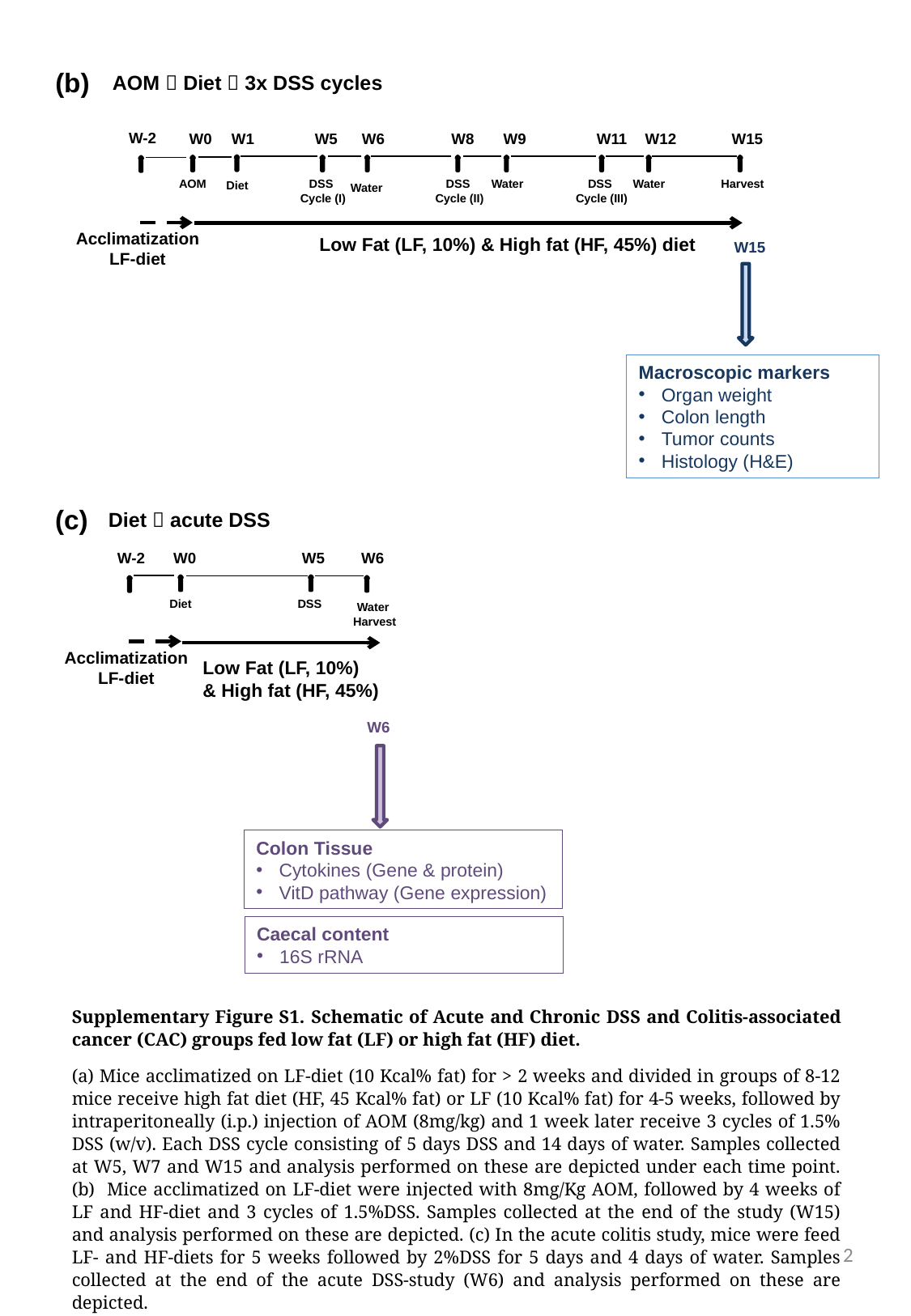

(b)
AOM  Diet  3x DSS cycles
W-2
W1
W0
W5
W6
W8
W9
W11
W12
W15
AOM
DSS
Cycle (I)
DSS
Cycle (II)
Water
DSS
Cycle (III)
Water
Harvest
Diet
Water
Acclimatization
LF-diet
Low Fat (LF, 10%) & High fat (HF, 45%) diet
W15
Macroscopic markers
Organ weight
Colon length
Tumor counts
Histology (H&E)
(c)
Diet  acute DSS
W0
W5
W6
W-2
Diet
DSS
Water
Harvest
Acclimatization
LF-diet
Low Fat (LF, 10%)
& High fat (HF, 45%)
W6
Colon Tissue
Cytokines (Gene & protein)
VitD pathway (Gene expression)
Caecal content
16S rRNA
Supplementary Figure S1. Schematic of Acute and Chronic DSS and Colitis-associated cancer (CAC) groups fed low fat (LF) or high fat (HF) diet.
(a) Mice acclimatized on LF-diet (10 Kcal% fat) for > 2 weeks and divided in groups of 8-12 mice receive high fat diet (HF, 45 Kcal% fat) or LF (10 Kcal% fat) for 4-5 weeks, followed by intraperitoneally (i.p.) injection of AOM (8mg/kg) and 1 week later receive 3 cycles of 1.5% DSS (w/v). Each DSS cycle consisting of 5 days DSS and 14 days of water. Samples collected at W5, W7 and W15 and analysis performed on these are depicted under each time point. (b) Mice acclimatized on LF-diet were injected with 8mg/Kg AOM, followed by 4 weeks of LF and HF-diet and 3 cycles of 1.5%DSS. Samples collected at the end of the study (W15) and analysis performed on these are depicted. (c) In the acute colitis study, mice were feed LF- and HF-diets for 5 weeks followed by 2%DSS for 5 days and 4 days of water. Samples collected at the end of the acute DSS-study (W6) and analysis performed on these are depicted.
2

## Slide 3
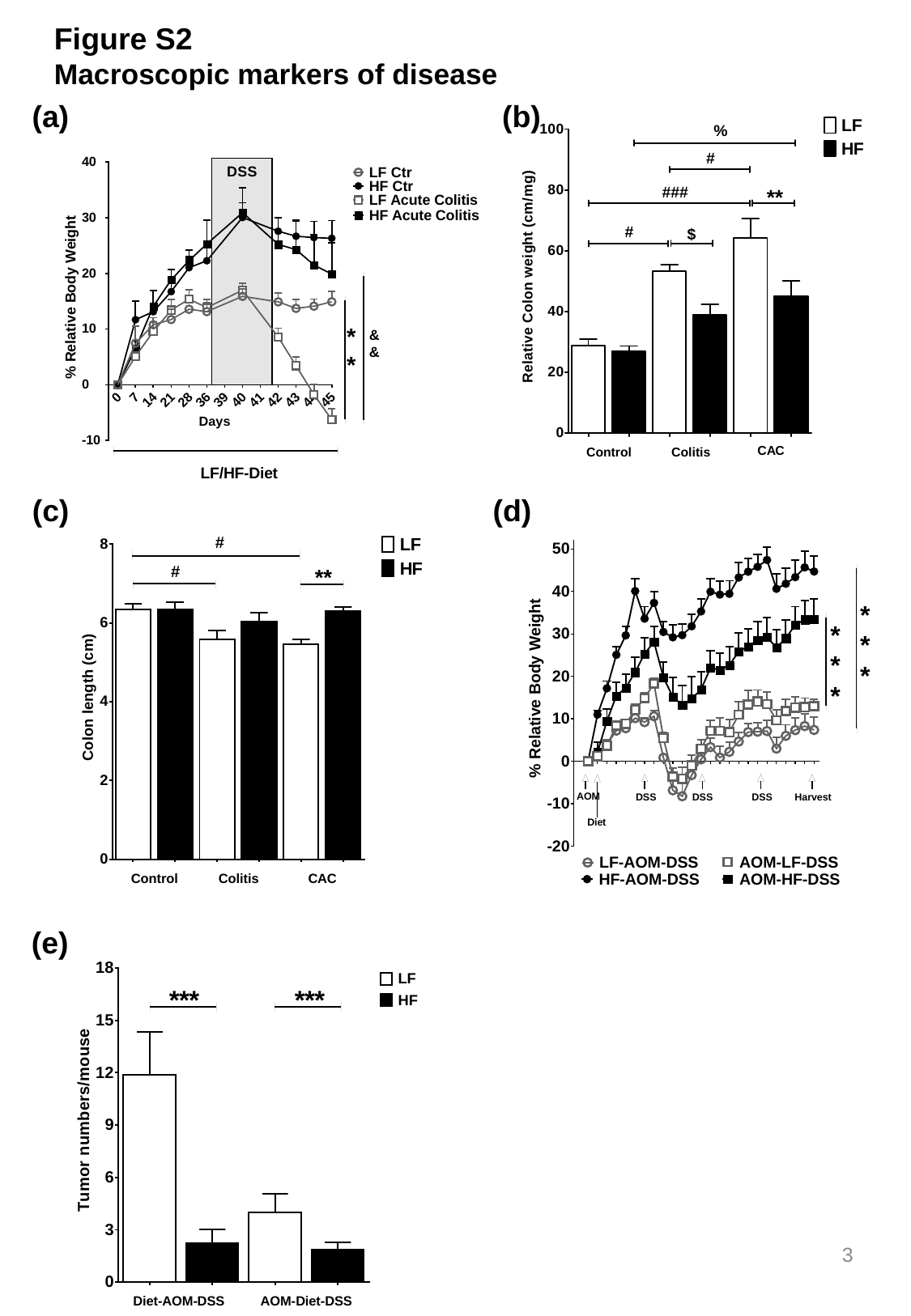

Figure S2
Macroscopic markers of disease
(b)
(a)
(d)
(c)
(e)
3

## Slide 4
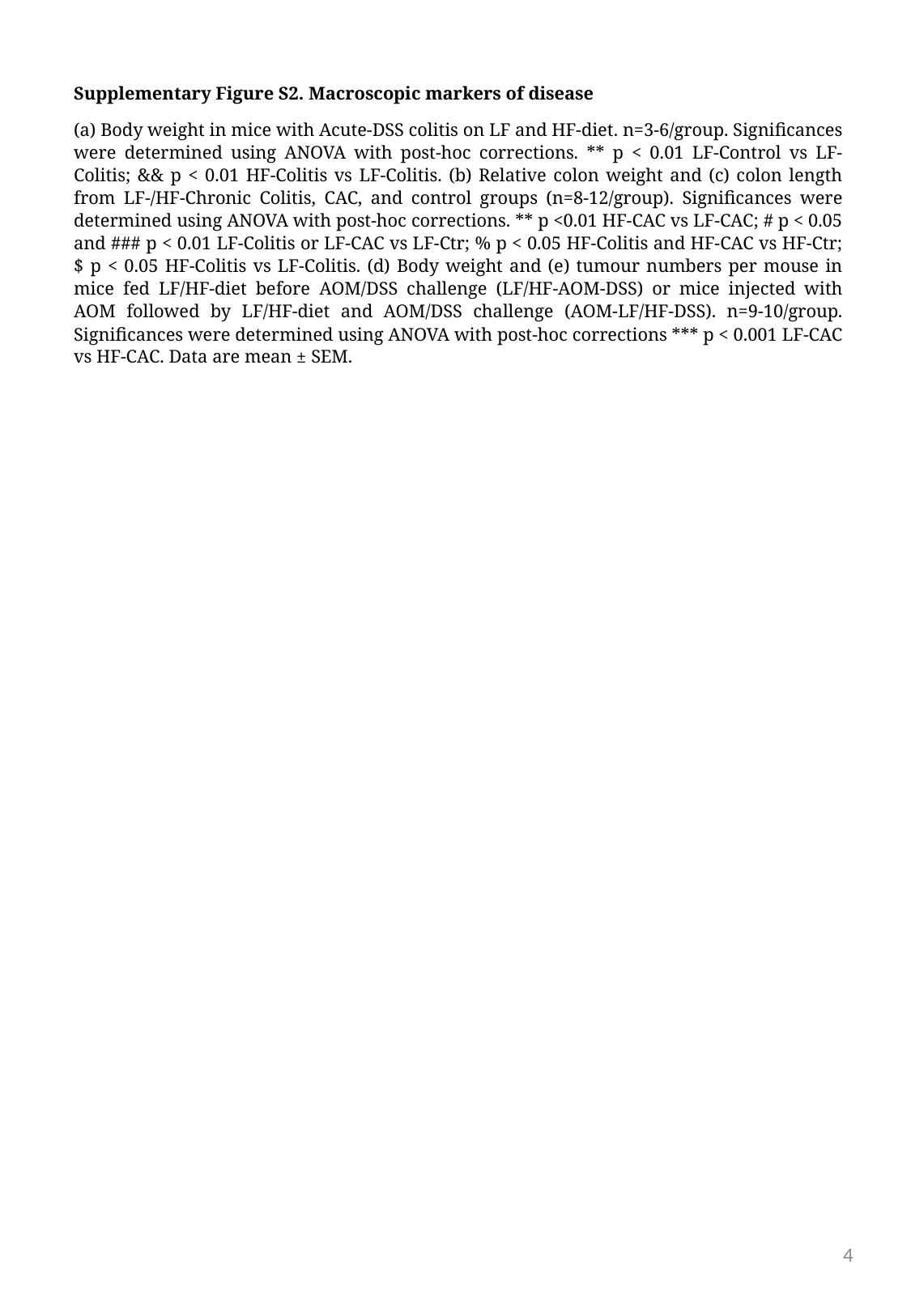

Supplementary Figure S2. Macroscopic markers of disease
(a) Body weight in mice with Acute-DSS colitis on LF and HF-diet. n=3-6/group. Significances were determined using ANOVA with post-hoc corrections. ** p < 0.01 LF-Control vs LF-Colitis; && p < 0.01 HF-Colitis vs LF-Colitis. (b) Relative colon weight and (c) colon length from LF-/HF-Chronic Colitis, CAC, and control groups (n=8-12/group). Significances were determined using ANOVA with post-hoc corrections. ** p <0.01 HF-CAC vs LF-CAC; # p < 0.05 and ### p < 0.01 LF-Colitis or LF-CAC vs LF-Ctr; % p < 0.05 HF-Colitis and HF-CAC vs HF-Ctr; $ p < 0.05 HF-Colitis vs LF-Colitis. (d) Body weight and (e) tumour numbers per mouse in mice fed LF/HF-diet before AOM/DSS challenge (LF/HF-AOM-DSS) or mice injected with AOM followed by LF/HF-diet and AOM/DSS challenge (AOM-LF/HF-DSS). n=9-10/group. Significances were determined using ANOVA with post-hoc corrections *** p < 0.001 LF-CAC vs HF-CAC. Data are mean ± SEM.
4

## Slide 5
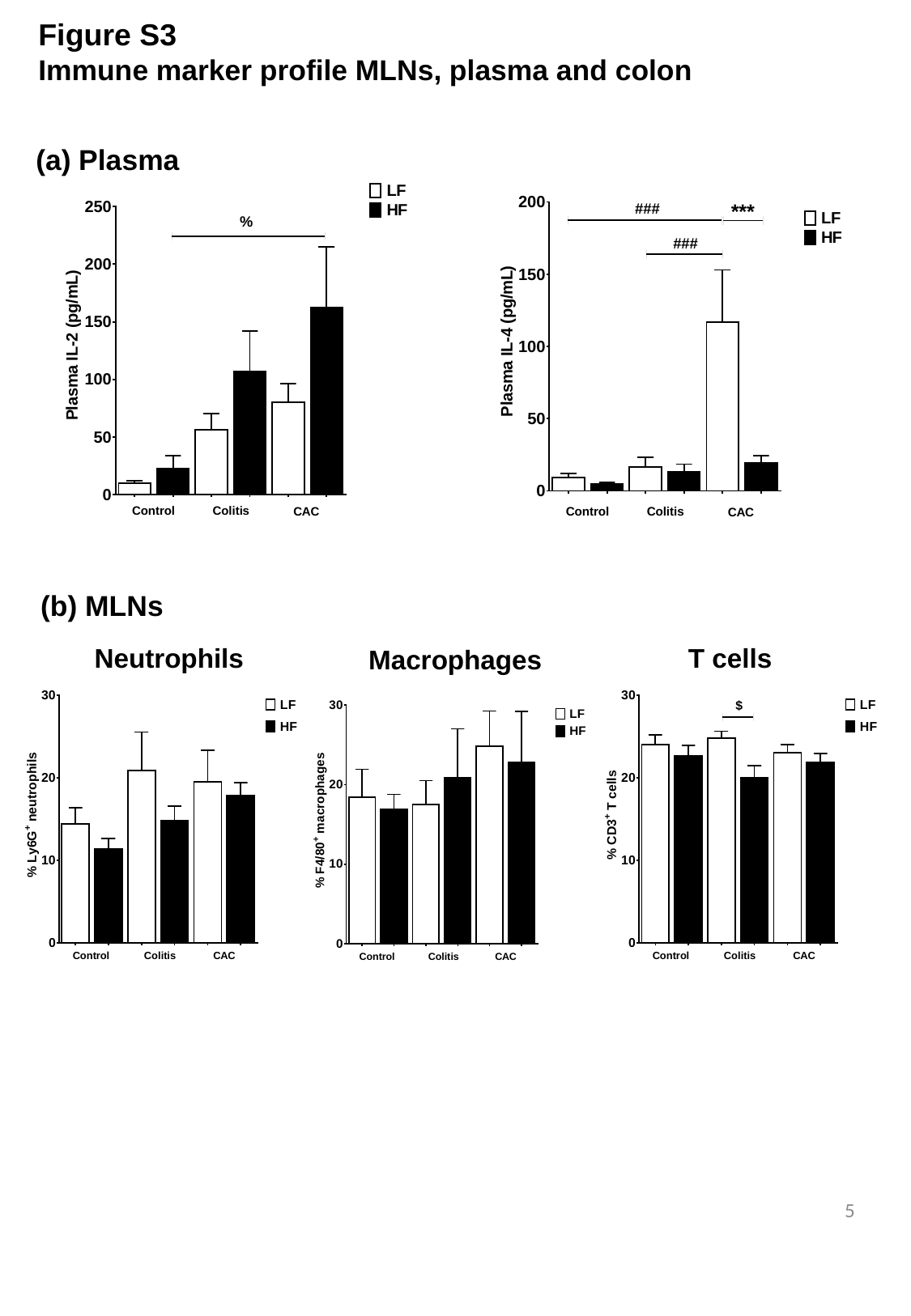

Figure S3
Immune marker profile MLNs, plasma and colon
(a) Plasma
(b) MLNs
Neutrophils
T cells
Macrophages
5

## Slide 6
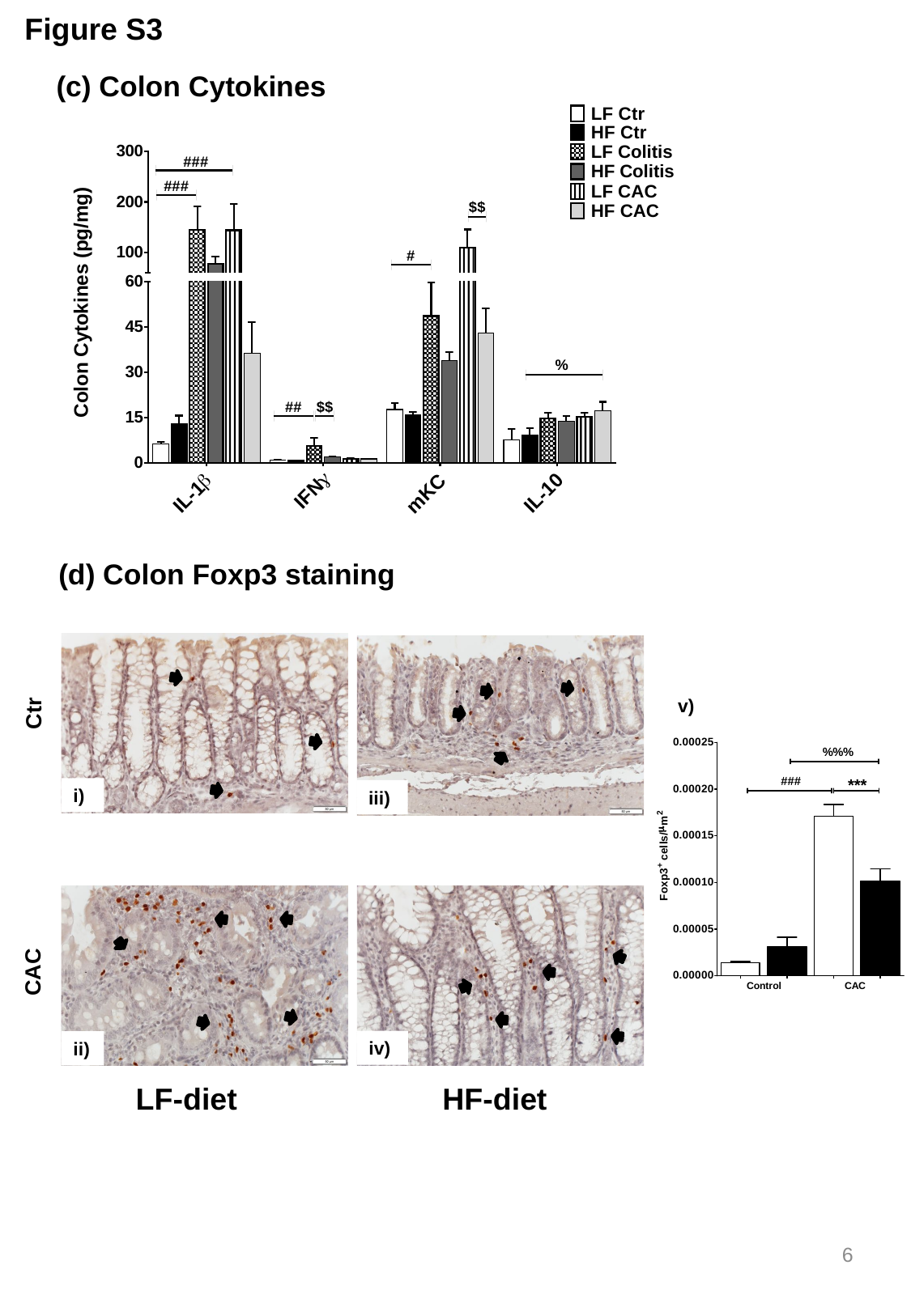

Figure S3
(c) Colon Cytokines
(d) Colon Foxp3 staining
Ctr
v)
i)
iii)
CAC
iv)
ii)
LF-diet
HF-diet
6

## Slide 7
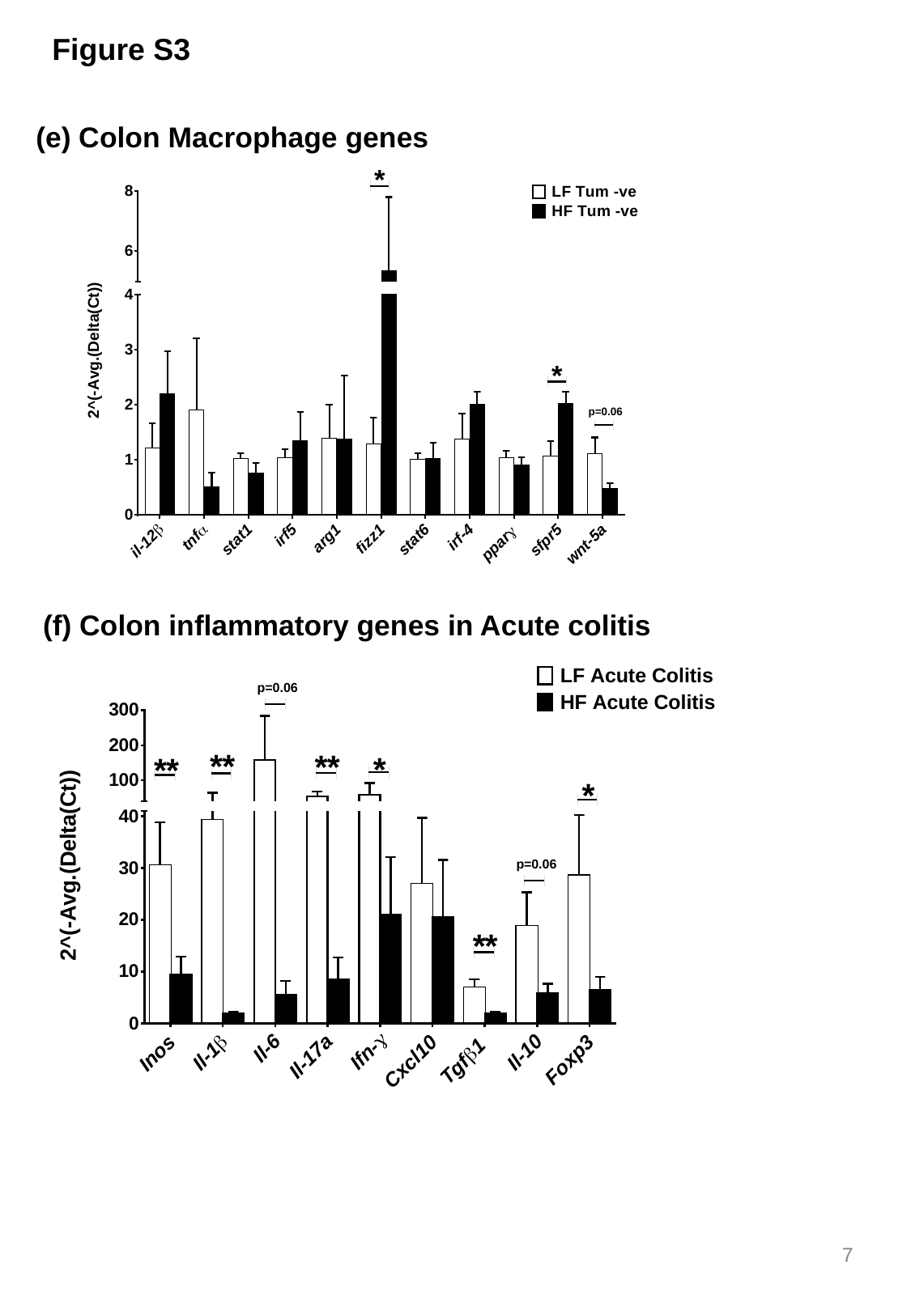

Figure S3
(e) Colon Macrophage genes
(f) Colon inflammatory genes in Acute colitis
7

## Slide 8
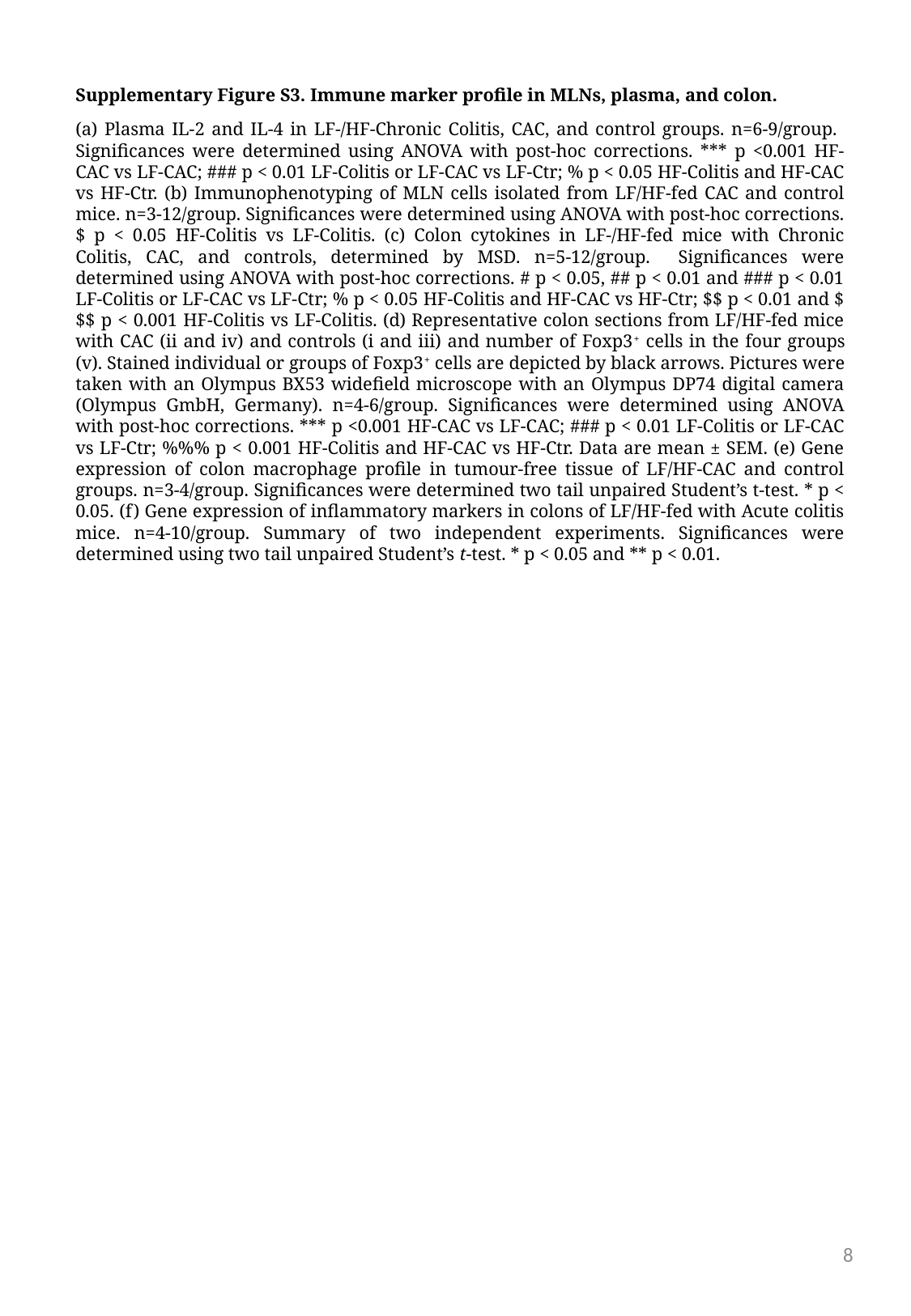

Supplementary Figure S3. Immune marker profile in MLNs, plasma, and colon.
(a) Plasma IL-2 and IL-4 in LF-/HF-Chronic Colitis, CAC, and control groups. n=6-9/group. Significances were determined using ANOVA with post-hoc corrections. *** p <0.001 HF-CAC vs LF-CAC; ### p < 0.01 LF-Colitis or LF-CAC vs LF-Ctr; % p < 0.05 HF-Colitis and HF-CAC vs HF-Ctr. (b) Immunophenotyping of MLN cells isolated from LF/HF-fed CAC and control mice. n=3-12/group. Significances were determined using ANOVA with post-hoc corrections. $ p < 0.05 HF-Colitis vs LF-Colitis. (c) Colon cytokines in LF-/HF-fed mice with Chronic Colitis, CAC, and controls, determined by MSD. n=5-12/group. Significances were determined using ANOVA with post-hoc corrections. # p < 0.05, ## p < 0.01 and ### p < 0.01 LF-Colitis or LF-CAC vs LF-Ctr; % p < 0.05 HF-Colitis and HF-CAC vs HF-Ctr; $$ p < 0.01 and $$$ p < 0.001 HF-Colitis vs LF-Colitis. (d) Representative colon sections from LF/HF-fed mice with CAC (ii and iv) and controls (i and iii) and number of Foxp3+ cells in the four groups (v). Stained individual or groups of Foxp3+ cells are depicted by black arrows. Pictures were taken with an Olympus BX53 widefield microscope with an Olympus DP74 digital camera (Olympus GmbH, Germany). n=4-6/group. Significances were determined using ANOVA with post-hoc corrections. *** p <0.001 HF-CAC vs LF-CAC; ### p < 0.01 LF-Colitis or LF-CAC vs LF-Ctr; %%% p < 0.001 HF-Colitis and HF-CAC vs HF-Ctr. Data are mean ± SEM. (e) Gene expression of colon macrophage profile in tumour-free tissue of LF/HF-CAC and control groups. n=3-4/group. Significances were determined two tail unpaired Student’s t-test. * p < 0.05. (f) Gene expression of inflammatory markers in colons of LF/HF-fed with Acute colitis mice. n=4-10/group. Summary of two independent experiments. Significances were determined using two tail unpaired Student’s t-test. * p < 0.05 and ** p < 0.01.
8

## Slide 9
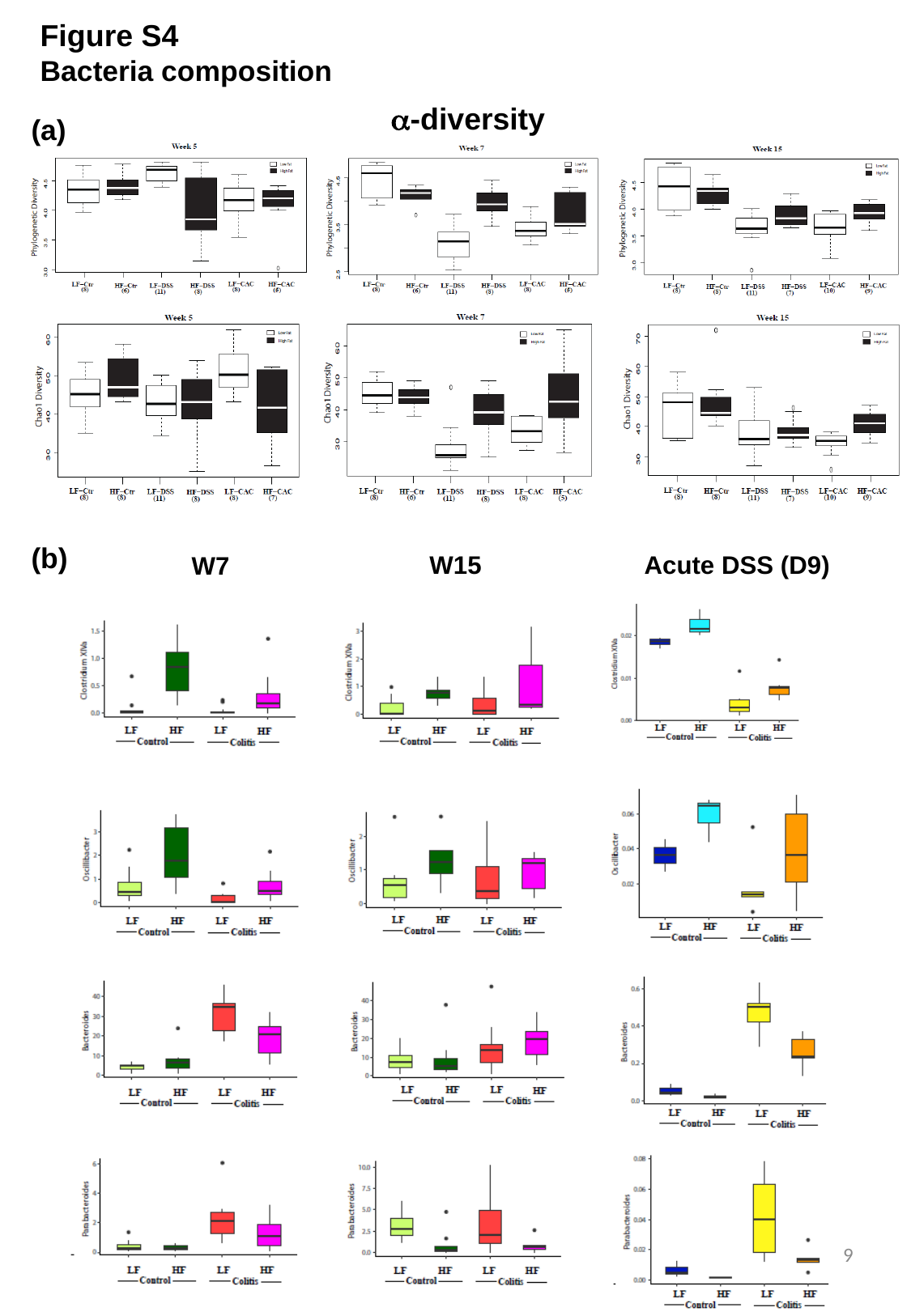

Figure S4
Bacteria composition
a-diversity
(a)
(b)
W15
Acute DSS (D9)
W7
9

## Slide 10
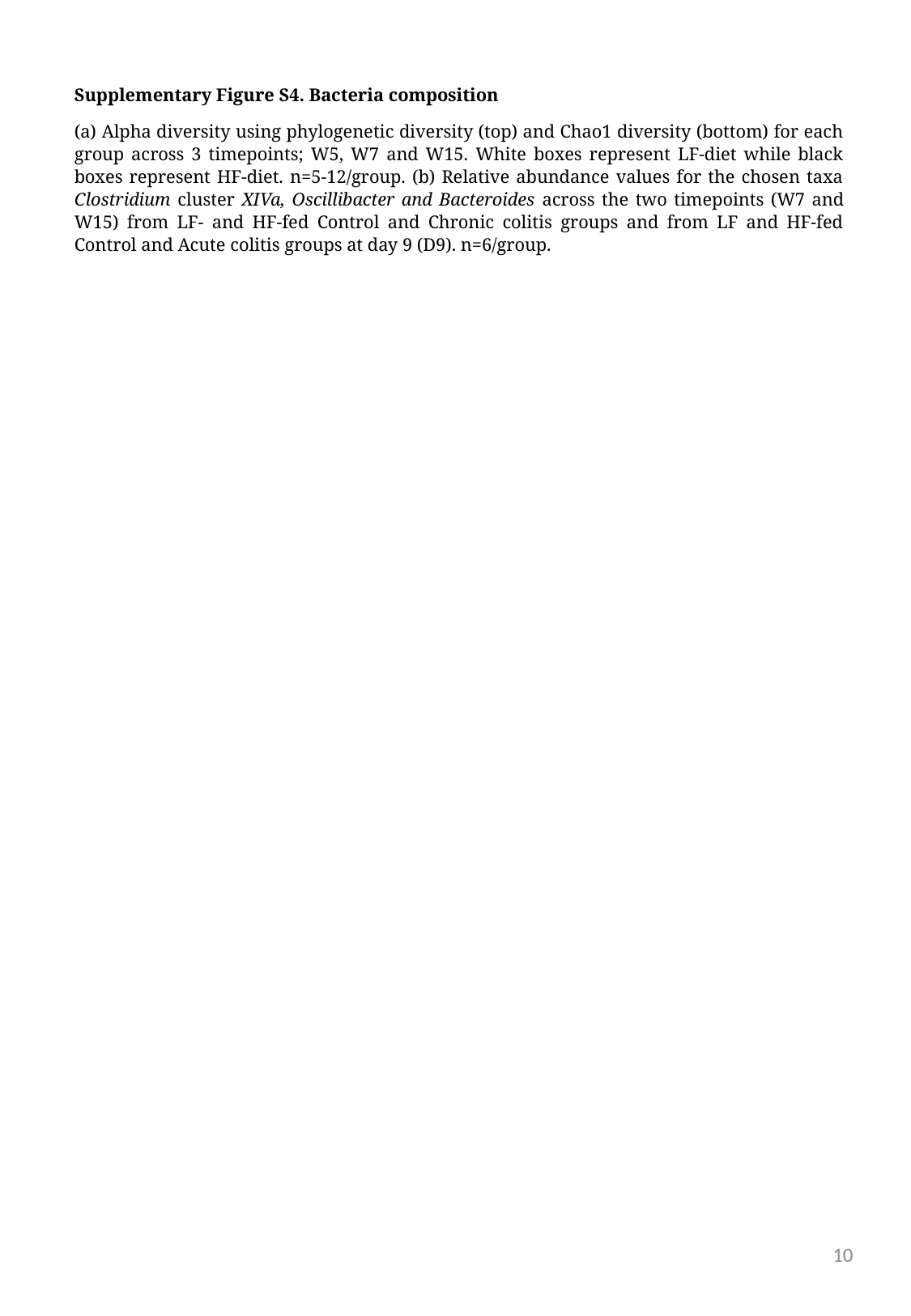

Supplementary Figure S4. Bacteria composition
(a) Alpha diversity using phylogenetic diversity (top) and Chao1 diversity (bottom) for each group across 3 timepoints; W5, W7 and W15. White boxes represent LF-diet while black boxes represent HF-diet. n=5-12/group. (b) Relative abundance values for the chosen taxa Clostridium cluster XIVa, Oscillibacter and Bacteroides across the two timepoints (W7 and W15) from LF- and HF-fed Control and Chronic colitis groups and from LF and HF-fed Control and Acute colitis groups at day 9 (D9). n=6/group.
10

## Slide 11
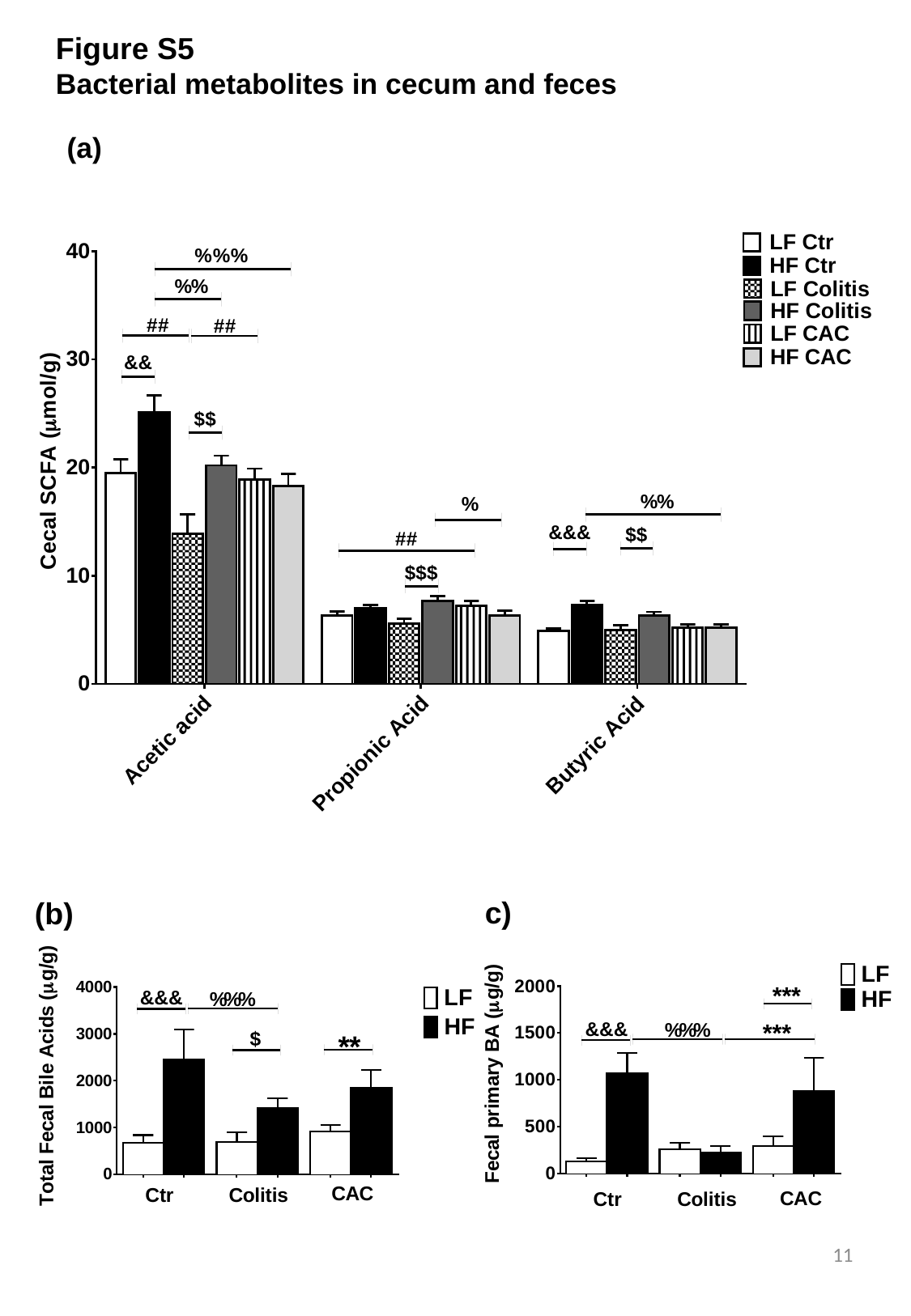

Figure S5
Bacterial metabolites in cecum and feces
(a)
c)
(b)
11

## Slide 12
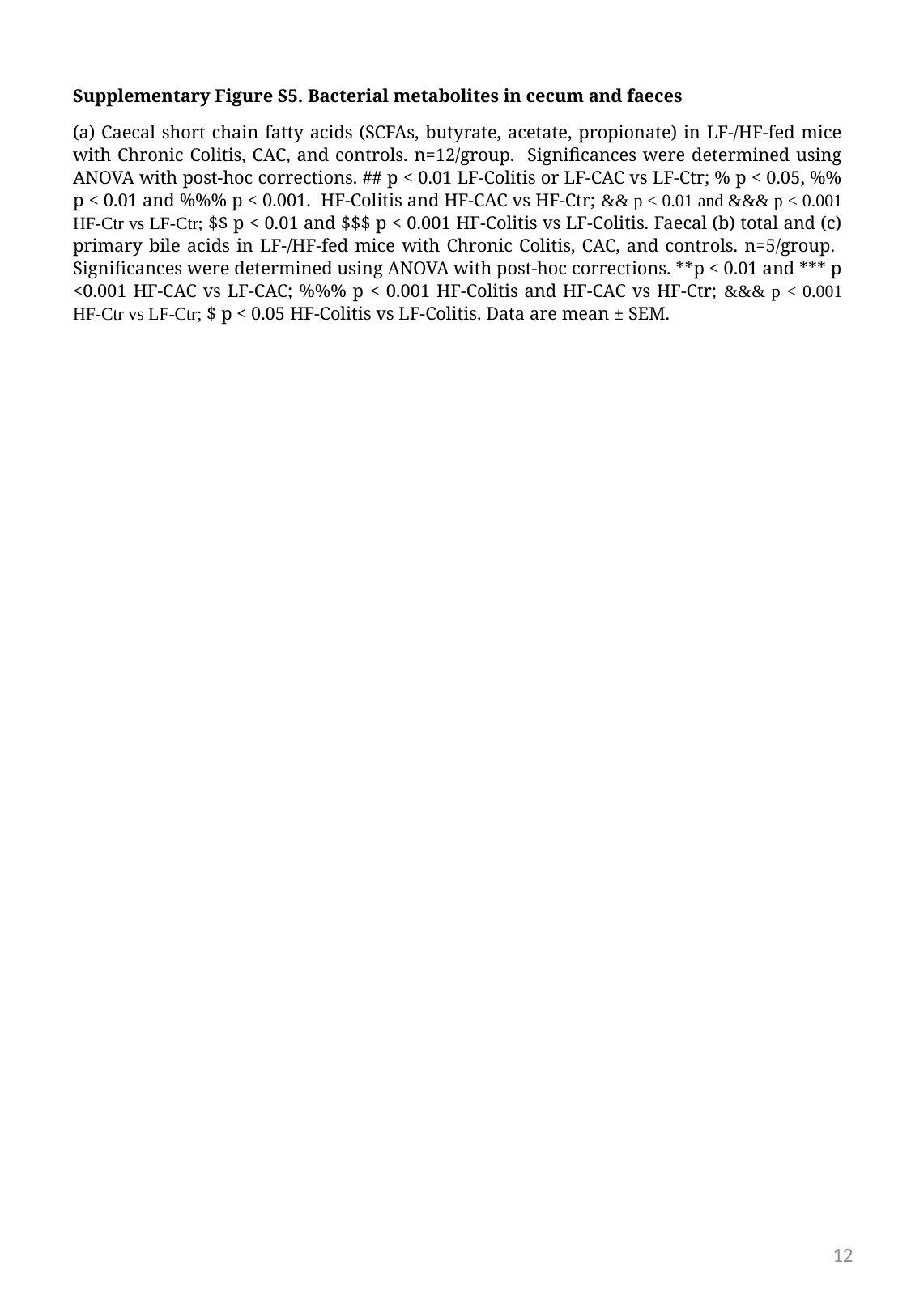

Supplementary Figure S5. Bacterial metabolites in cecum and faeces
(a) Caecal short chain fatty acids (SCFAs, butyrate, acetate, propionate) in LF-/HF-fed mice with Chronic Colitis, CAC, and controls. n=12/group. Significances were determined using ANOVA with post-hoc corrections. ## p < 0.01 LF-Colitis or LF-CAC vs LF-Ctr; % p < 0.05, %% p < 0.01 and %%% p < 0.001. HF-Colitis and HF-CAC vs HF-Ctr; && p < 0.01 and &&& p < 0.001 HF-Ctr vs LF-Ctr; $$ p < 0.01 and $$$ p < 0.001 HF-Colitis vs LF-Colitis. Faecal (b) total and (c) primary bile acids in LF-/HF-fed mice with Chronic Colitis, CAC, and controls. n=5/group. Significances were determined using ANOVA with post-hoc corrections. **p < 0.01 and *** p <0.001 HF-CAC vs LF-CAC; %%% p < 0.001 HF-Colitis and HF-CAC vs HF-Ctr; &&& p < 0.001 HF-Ctr vs LF-Ctr; $ p < 0.05 HF-Colitis vs LF-Colitis. Data are mean ± SEM.
12

## Slide 13
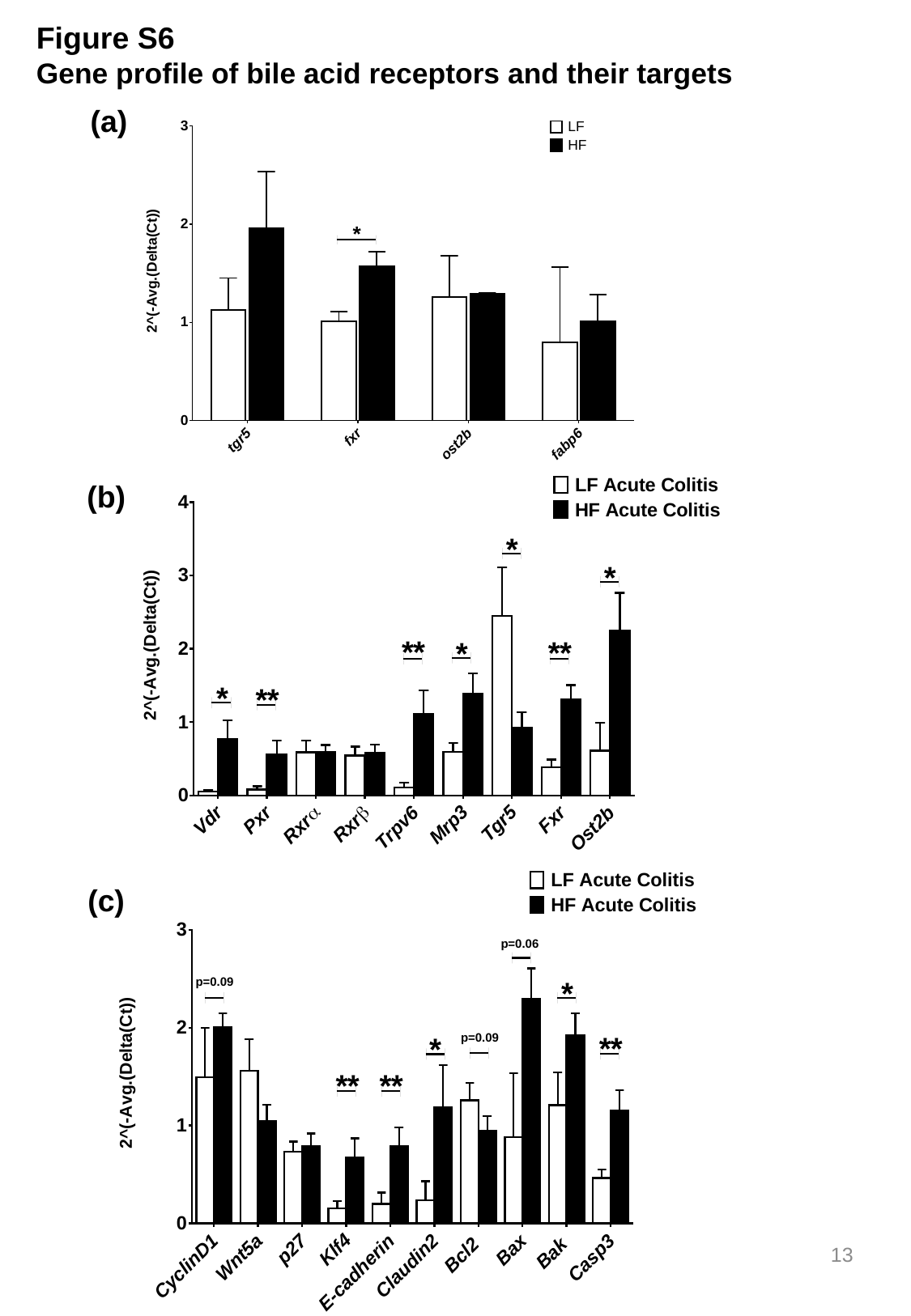

Figure S6
Gene profile of bile acid receptors and their targets
(a)
(b)
(c)
13

## Slide 14
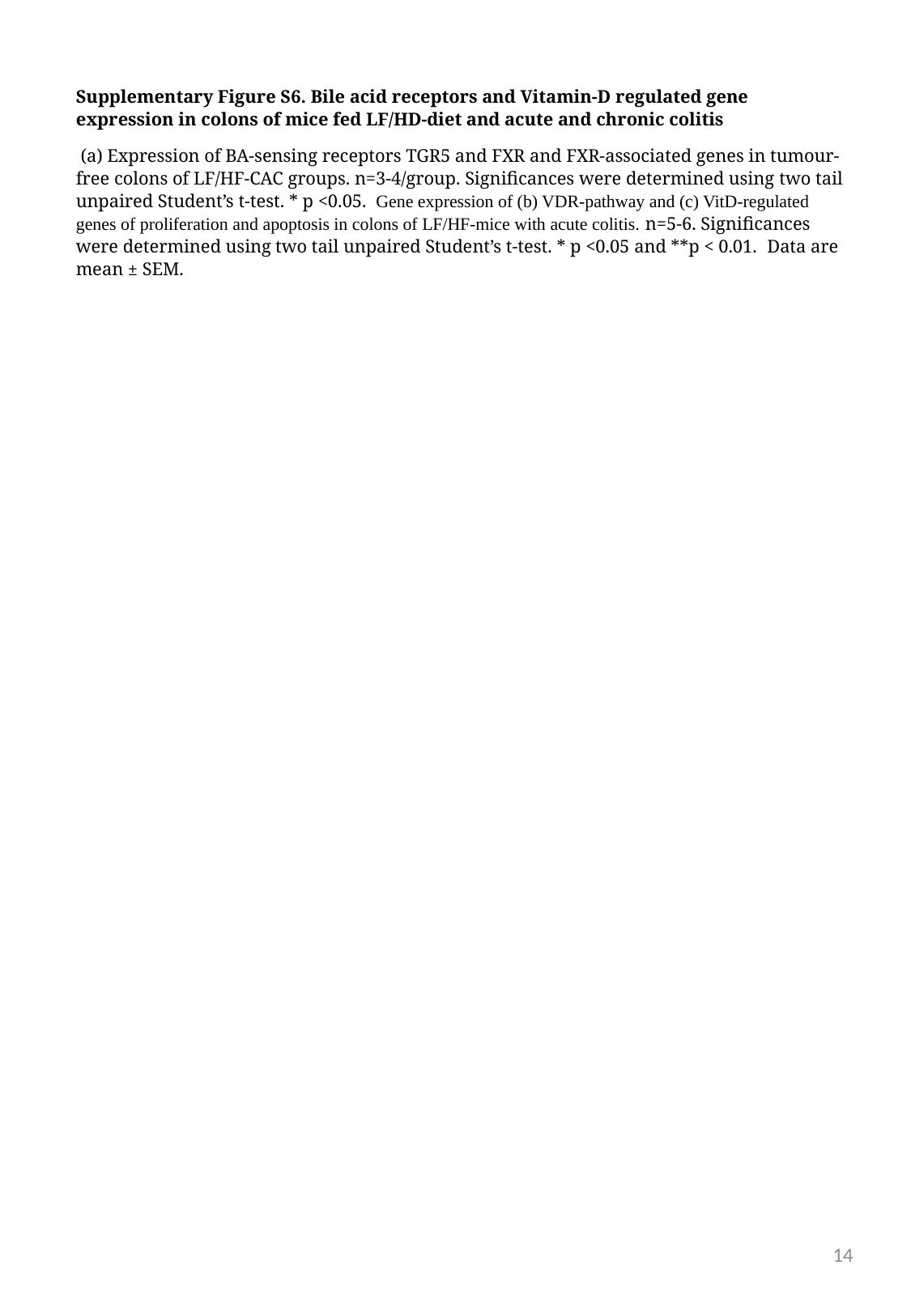

Supplementary Figure S6. Bile acid receptors and Vitamin-D regulated gene expression in colons of mice fed LF/HD-diet and acute and chronic colitis
 (a) Expression of BA-sensing receptors TGR5 and FXR and FXR-associated genes in tumour-free colons of LF/HF-CAC groups. n=3-4/group. Significances were determined using two tail unpaired Student’s t-test. * p <0.05. Gene expression of (b) VDR-pathway and (c) VitD-regulated genes of proliferation and apoptosis in colons of LF/HF-mice with acute colitis. n=5-6. Significances were determined using two tail unpaired Student’s t-test. * p <0.05 and **p < 0.01. Data are mean ± SEM.
14

## Slide 15
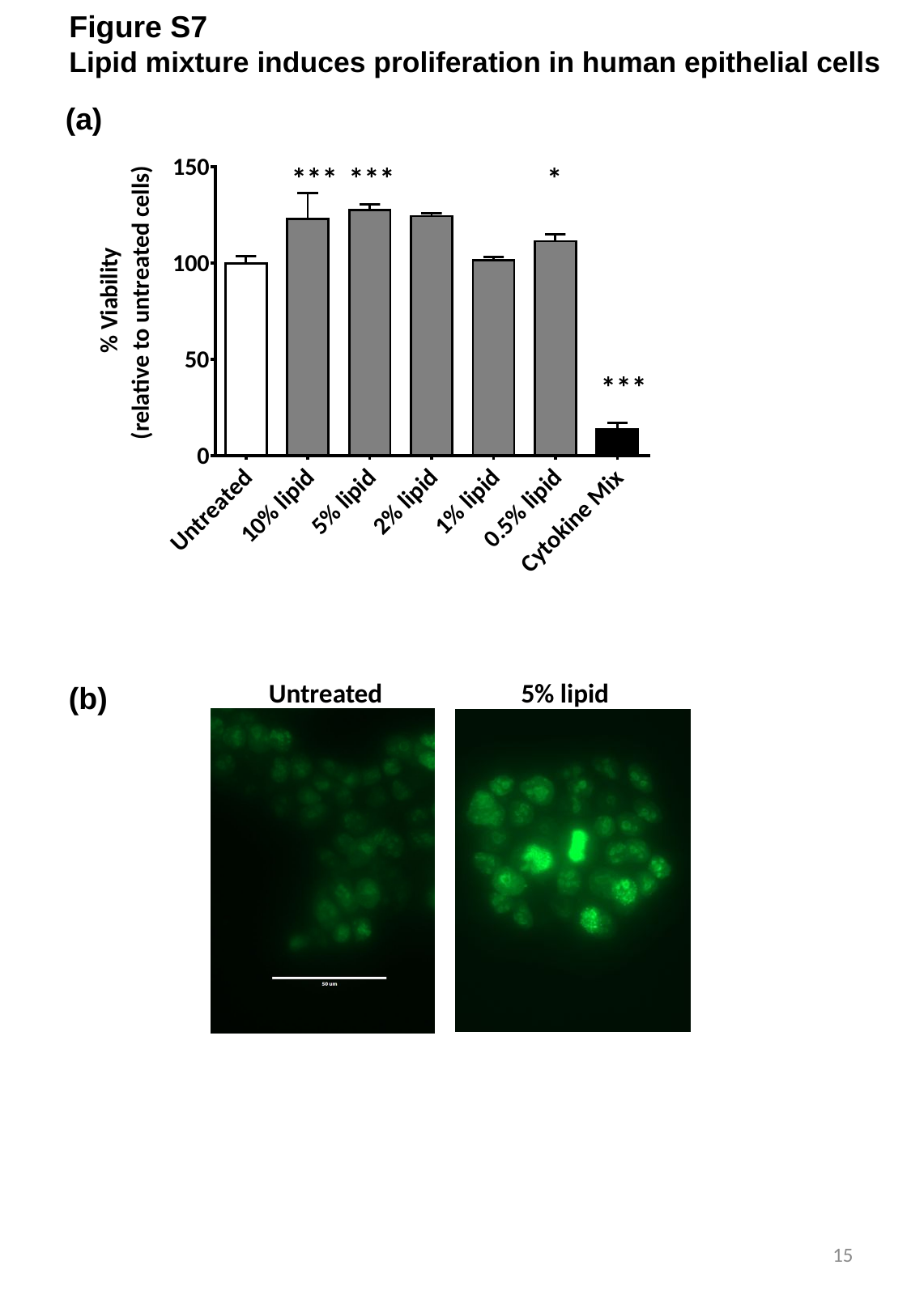

Figure S7
Lipid mixture induces proliferation in human epithelial cells
(a)
Untreated
5% lipid
(b)
15

## Slide 16
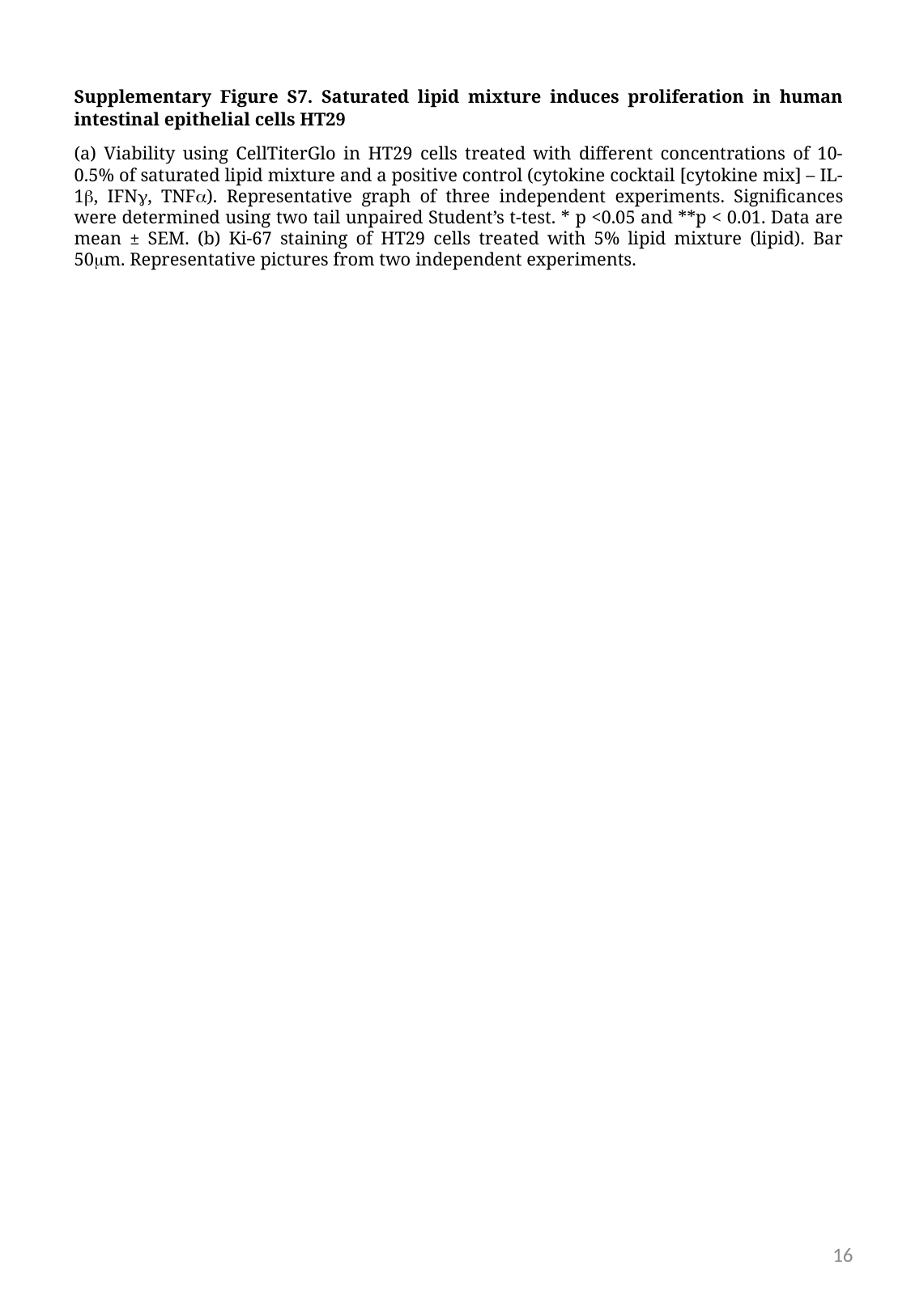

Supplementary Figure S7. Saturated lipid mixture induces proliferation in human intestinal epithelial cells HT29
(a) Viability using CellTiterGlo in HT29 cells treated with different concentrations of 10-0.5% of saturated lipid mixture and a positive control (cytokine cocktail [cytokine mix] – IL-1b, IFNg, TNFa). Representative graph of three independent experiments. Significances were determined using two tail unpaired Student’s t-test. * p <0.05 and **p < 0.01. Data are mean ± SEM. (b) Ki-67 staining of HT29 cells treated with 5% lipid mixture (lipid). Bar 50mm. Representative pictures from two independent experiments.
16
